# REUNION: transcription factor binding prediction and regulatory association inference from single-cell multi-omics data

**DOI:** 10.1093/bioinformatics/btae234

**Published:** 2024-06-28

**Authors:** Yang Yang, Dana Pe’er

**Affiliations:** Computational and Systems Biology Program, Sloan Kettering Institute, Memorial Sloan Kettering Cancer Center, New York, NY 10065, United States; Howard Hughes Medical Institute, Chevy Chase, MD 20815, United States; Computational and Systems Biology Program, Sloan Kettering Institute, Memorial Sloan Kettering Cancer Center, New York, NY 10065, United States; Howard Hughes Medical Institute, Chevy Chase, MD 20815, United States

## Abstract

**Motivation:**

Profiling of gene expression and chromatin accessibility by single-cell multi-omics approaches can help to systematically decipher how transcription factors (TFs) regulate target gene expression via *cis*-region interactions. However, integrating information from different modalities to discover regulatory associations is challenging, in part because motif scanning approaches miss many likely TF binding sites.

**Results:**

We develop REUNION, a framework for predicting genome-wide TF binding and *cis*-region-TF-gene “triplet” regulatory associations using single-cell multi-omics data. The first component of REUNION, Unify, utilizes information theory-inspired complementary score functions that incorporate TF expression, chromatin accessibility, and target gene expression to identify regulatory associations. The second component, Rediscover, takes Unify estimates as input for pseudo semi-supervised learning to predict TF binding in accessible genomic regions that may or may not include detected TF motifs. Rediscover leverages latent chromatin accessibility and sequence feature spaces of the genomic regions, without requiring chromatin immunoprecipitation data for model training. Applied to peripheral blood mononuclear cell data, REUNION outperforms alternative methods in TF binding prediction on average performance. In particular, it recovers missing region-TF associations from regions lacking detected motifs, which circumvents the reliance on motif scanning and facilitates discovery of novel associations involving potential co-binding transcriptional regulators. Newly identified region-TF associations, even in regions lacking a detected motif, improve the prediction of target gene expression in regulatory triplets, and are thus likely to genuinely participate in the regulation.

**Availability and implementation:**

All source code is available at https://github.com/yangymargaret/REUNION.

## 1 Introduction

Single-cell multi-omics technologies can simultaneously profile chromatin state and RNA from the same cell, and represent an exciting opportunity to align transcriptional and epigenomic views of the genome to explore the gene regulation underlying cell fate decisions (Cao *et al.* 2018, Chen *et al.* 2019, Ma *et al.* 2020, Wang *et al.* 2021). However, the combinatorial complexity of gene regulation, combined with the substantial noise and sparsity of the data, present computational challenges that have not been sufficiently addressed by regulatory inference algorithms. Transcription factors (TFs), regulatory sequence elements within accessible genomic regions, and target genes are key components of gene regulatory networks (GRNs). Typically, assay for transposase-accessible chromatin with sequencing (ATAC-seq) peaks are used to denote accessible regions. By associating “triplets” of a specific chromatin accessibility peak or region, a putative TF that binds it, and the gene under regulatory control, we can improve GRN inference and better understand gene regulation in development and disease. However, existing methods usually fail to capture the interplay between these components. Most only utilize two feature types to estimate pairwise region-TF, region-gene or TF-gene associations separately (Ma *et al.* 2020, Stuart *et al.* 2021, Argelaguet et al. 2022, Bravo González-Blas *et al.* 2023, Kamal *et al.* 2023, Kartha *et al.* 2022). Some further assemble the pairwise associations into region-TF-gene links (Bravo González-Blas *et al.* 2023, Kamal *et al.* 2023), whereas others infer GRNs without constructing triplet links (Ma *et al.* 2020, Argelaguet et al. 2022, Kartha *et al.* 2022). As pairwise associations may also depend on the third feature type, considering only two may lose critical information. Two recent methods, Pando (Fleck *et al.* 2023) and TRIPOD (Jiang *et al.* 2022), estimate peak-TF-gene associations by utilizing information from all three modalities. Pando predicts target gene expression by linear regression using the product of TF expression and peak accessibility. TRIPOD performs two tests of conditional association by matching cell aggregates by peak accessibility or TF expression. Both methods have high time complexity and limited recall (see Supplementary Material).

Most single-cell multi-omics analysis methods establish peak-TF links based on TF binding motif detection within chromatin accessibility peaks, and mainly vary by which motif collection and motif scanning algorithm they use. However, this approach has several limitations. First, many TFs share similar binding motifs, which may lead to false positive peak-TF links and may undermine the validity of motif enrichment analysis (often used to identify TF regulators) (Ma *et al.* 2020). Second, motif presence may not correspond to active TF binding events. For example, the TF may not be expressed in the cell. To address this, the *in silico* ChIP-seq library method (Argelaguet et al. 2022) utilizes the correlation between TF expression and the putative target peak accessibility to compute a regularized TF binding score. A similar approach is used in GRaNIE (Kamal *et al.* 2023). However, both methods have difficulty in capturing TF binding sites (TFBSs) if TF expression is only weakly correlated with peak accessibility. There are TF footprinting-based methods to predict TF binding from bulk ATAC-seq data (Li *et al.* 2019b, Bentsen *et al.* 2020), which also rely on motif detection, but these do not infer gene associations. Additional challenges of motif-based approaches are that motif scanning largely relies on sequences alone and may miss many motifs; motif databases are incomplete; and TFs cannot be linked to accessible regions that lack detected TF motifs. The missing peak-TF links may limit the accurate prediction of gene expression, discovery of combinatorial TF regulation, and evaluation of other potential links.

Current single-cell multi-omics methods do not venture beyond motif scanning to recover potential peak-TF links from peaks lacking known motifs. In contrast, many supervised learning approaches have been developed to predict TF binding utilizing DNA sequence features in combination with epigenomic data (Keilwagen *et al.* 2019, Quang and Xie 2019, Li *et al.* 2019a, Fu *et al.* 2020, Chen *et al.* 2021, Cazares *et al.* 2023). These methods generally require ChIP-seq (chromatin immunoprecipitation with sequencing) data for training, which are costly to obtain and only available for a few TFs in limited biological contexts or cell types.

Here, we integrate feature types in single-cell multi-omics data to improve the prediction of TF binding to genomic regions. We first infer triplet peak-TF-gene associations to ensure more accurate peak-TF calls within accessible regions, and then use pseudo semi-supervised learning to recover potential missed peak-TF associations, particularly in regions that lack known motifs. Our approach captures more binding interactions than alternative methods, at higher specificity.

## 2 Approach

**Figure 1.**
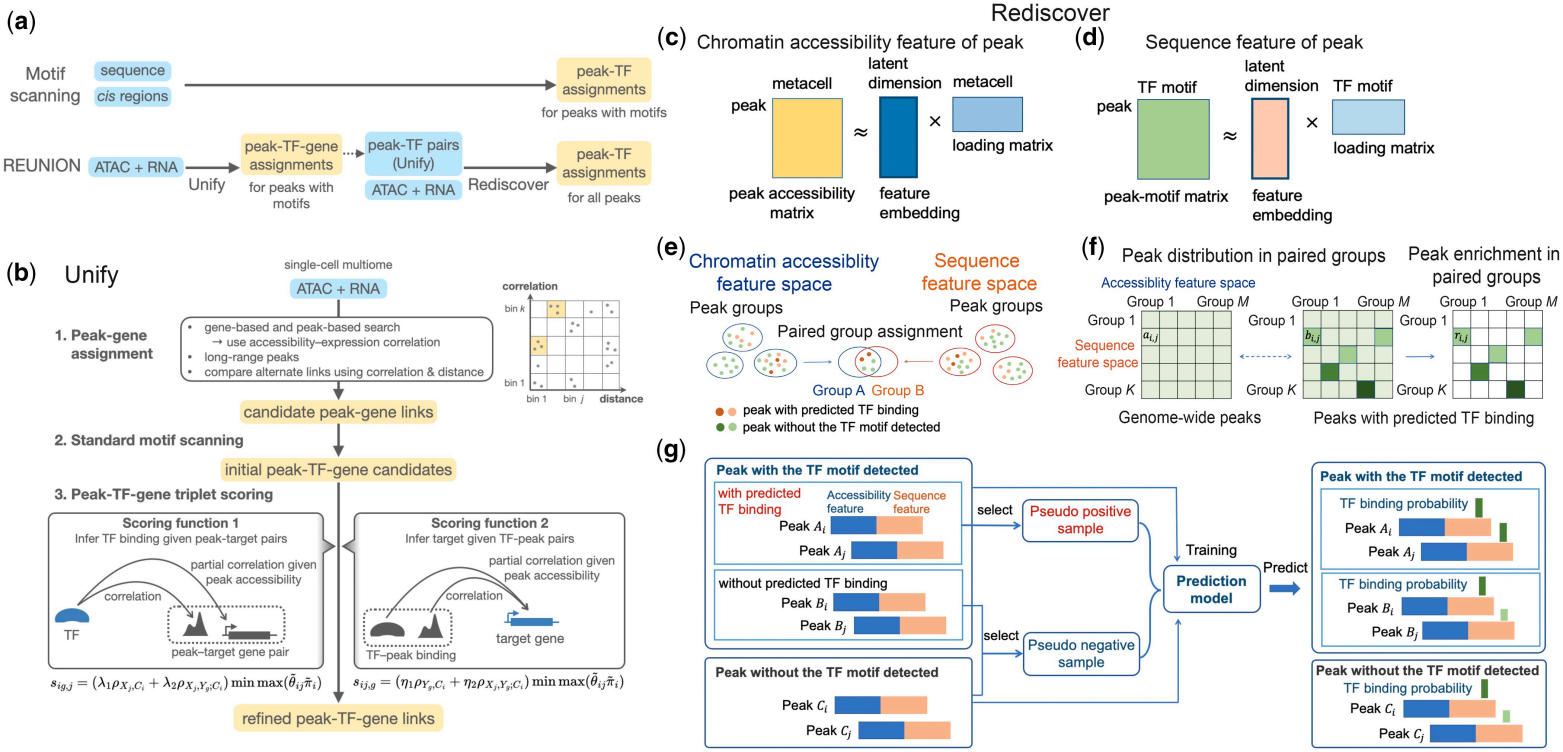
Schematic overview of REUNION. (a) Motif scanning approaches only identify peak-TF associations in chromatin regions that harbor predicted motifs, often relying on sequences alone. REUNION first applies Unify to single-cell multiome data to infer robust peak-TF-gene triplets in chromatin accessibility peaks with predicted motifs. It then takes peak-TF pairs from Unify as input for Rediscover, which uses pseudo semi-supervised learning to recover missed peak-TF associations from all accessibility peaks, including those lacking detected motifs. (b) Unify includes three successive modules for (i) assigning accessibility peaks to genes, (ii) standard TF motif scanning, and (iii) evaluating the strength of possible peak-TF-gene triplet associations using complementary score functions. The use of triplet information generates more specific peak-TF predictions than motif scanning alone. (c–g) The modules of Rediscover. SVD is used to factorize the peak accessibility and peak-motif matrices to generate representations of peaks using lower-dimensional embeddings of chromatin accessibility features (c) and motif-based sequence features (d), respectively, followed by peak clustering in each feature space (e). In (e) the darker dots represent peaks with membership in both groups. (f) Enrichment analysis of peaks in the paired groups. (g) Pseudo-labeled training sample selection and prediction model training.

We developed the computational framework REUNION, which uses single-cell multi-omics (RNA + ATAC) data to infer peak-TF-gene triplet regulatory associations, and detects TF activity in a manner that is not constrained by motif scanning results. REUNION consists of the modular methods Unify and Rediscover, applied in two successive steps (Fig. 1a).

Unify identifies peak-TF-gene triplet regulatory associations from single-cell multiome data (Fig. 1a). It first assigns (near or distant) peaks to genes utilizing both distance constraints and the correlation between chromatin accessibility and gene expression, and uses standard motif scanning to initially predict TF binding within these regions. The algorithm then applies two complementary score functions, inspired by information theory, that combine TF expression, peak accessibility, and target expression to assess the strength of each triplet. The use of triplet information (ensuring that a TF acts in a given peak to regulate its associated target gene) rather than paired variables is a key feature of Unify, aimed at reducing false associations.

Rediscover accepts Unify peak-TF link estimates as input for pseudo semi-supervised learning (Fig. 1c–g). We assume that peaks bound by a specific TF have distinctive chromatin accessibility and sequence patterns that can be used to discriminate them from unbound peaks. The Rediscover classifier thus leverages two integrated latent spaces with embedded chromatin and sequence features that effectively model TF-defined cell states. Critically, this enables it to refine Unify predictions and to recover peak-TF associations across all peaks, regardless of whether they include TF motifs detected by motif scanning, without the need for chromatin immunoprecipitation data for training. Rediscover can also be run independently, using any peak-TF link predictions.

## 3 Materials and methods

REUNION incorporates Unify and Rediscover, which are described below and explained in greater detail in the Supplementary Material.

### 3.1 Unify for triplet regulatory association inference

Unify integrates two complementary score functions that jointly quantify the strength of each candidate peak-TF-gene triplet regulatory associations. Score function 1 predicts TF binding given the putative peak-gene link, while score function 2 infers the target gene given the putative peak-TF link (Fig. 1a). Both scores are conceptually inspired by the joint mutual information (JMI) (Bennasar *et al.* 2015) between the different features. In practice, it is challenging to estimate the JMI score for continuous variables from limited data with noise and sparsity. Moreover, JMI is nonnegative and does not indicate positive or negative regulation. We thus use correlation and partial correlation instead of the mutual information (MI) and conditional mutual information (CMI) scores that form the JMI, respectively. Partial correlation quantifies the strength and direction of the association between two random variables, with the effect of a given set of other observed variables removed. We specifically use Spearman’s rank correlation or partial correlation, which can capture the nonlinear relationships between the random variables.

(1) Score function 1 f(TF;(peak,gene)) estimates the shared information between the TF and the putative peak-gene pair, aiming at predicting TF binding by not only using the peak-TF correlation, but also incorporating information from the potential target gene of the peak. Suppose *X_j_*, *C_i_*, *Y_g_* represent the expression of TF *j*, accessibility of peak *i* (which contains the motif of TF *j*), and expression of the potential target gene *g*, respectively. In its information theoretic formulation, Score function 1 is $I(X_{j};C_{i},Y_{g})=I(X_{j},C_{i})+I(X_{j},Y_{g}|C_{i})$. $I(X_{j},C_{i})$ and $I(X_{j},Y_{g}|C_{i})$ represent the MI between *X_j_* and *C_i_*, and the CMI between *X_j_* and *Y_g_* conditioned on *C_i_*, respectively. We use peak accessibility-TF expression correlation ($\rho_{X_{j},C_{i}}$) and the partial correlation between target gene and TF expressions conditioned on the peak accessibility ($\rho_{X_{j},Y_{g};C_{i}}$) as alternatives to the MI and CMI scores, respectively.

Incorporating a regularization term (Supplementary Material), score 1 (the TF binding score of TF *j* in peak locus *i* with potential target gene *g*) is estimated as:

$$s_{ig,j}=(\lambda_{1}\rho_{X_{j},C_{i}}+\lambda_{2}\rho_{X_{j},Y_{g};C_{i}})\min\max({\tilde{\theta }}_{ij}{\tilde{\pi }}_{i}),$$

where ${\tilde{\theta }}_{ij},\;{\tilde{\pi }}_{i}$ represent the normalized motif score of TF motif *j* in peak *i*, and the transformed maximal accessibility of peak *i* in the metacells, respectively (Supplementary Material). minmax(·) represents min-max scaling.

(2) Score function 2 f((peak,TF);gene) estimates the shared information between the putative peak-TF pair and the potential target gene, in order to infer if the gene is a target of the paired peak and TF. We have

$$s_{ij,g}=(\eta_{1}\rho_{Y_{g},C_{i}}+\eta_{2}\rho_{X_{j},Y_{g};C_{i}})\;\min\;\max({\tilde{\theta }}_{ij}{\tilde{\pi }}_{i}),$$

which integrates the correlation between peak accessibility and the potential target gene expression ($\rho_{Y_{g},C_{i}}$), the partial correlation $\rho_{X_{j},Y_{g};C_{i}}$, and a regularization term. *λ*_1_, *λ*_2_, *η*_1_, *η*_2_ are adjustable weights, for which we use 0.5 or −0.5 depending on the inferred type of association (activation or repression).

To compute the two scores, Unify harbors a peak-gene assignment module, which performs hybrid search by (i) seeking the regulatory peaks of each target gene and (ii) identifying alternative potential target genes for each candidate peak, and then compares alternative links to reduce ambiguous associations and facilitate more reliable distal regulatory peak search (Fig. 1a) (Supplementary Material). The peak-TF links are initialized by TF motif scanning in the peak sequences to establish the candidate peak-TF-gene links. We used the motifmatchr package (Schep 2023) with the curated motif collection from the CIS-BP database (Weirauch *et al.* 2014, Schep *et al.* 2017) for motif scanning, using the recommended threshold *P*-value <5e−05 (Schep *et al.* 2017).

### 3.2 Pseudo semi-supervised learning of TF binding

Rediscover is a pseudo semi-supervised learning approach for predicting TF binding events in accessible genomic regions, including those not predicted to harbor TF binding motifs by motif scanning analysis. The crux of our approach is to learn a classifier on latent feature spaces that effectively model which cell states the TF defines, and what other TFs it tends to partner with. We assume that our latent space includes features that are informative for TF binding, making it more amenable to training a good classifier.

In our semi-supervised paradigm, we aim to learn a prediction model utilizing both labeled data (in our case, peaks with known binding status of a given TF) and unlabeled data. However, since single-cell multiome data does not provide ground truth information about TF binding, we lack actual labeled data. Instead, we resort to taking the high-confidence peak-TF link predictions from Unify (Fig. 1a and b) as pseudo-labeled samples likely to be enriched with true positives, and use these to train a binding prediction model for the TF. Unlike standard semi-supervised settings where at least some labeled data are provided, we face the added challenge of how to select the most accurate pseudo-labeled training data.

#### 3.2.1 Feature representation of peaks utilizing chromatin accessibility and sequence features

We hypothesize that peaks bound by a specific TF have distinctive chromatin accessibility and sequence patterns that can be used to discriminate them from unbound peaks.

(1) *Chromatin accessibility feature representation.* Peaks bound by a given TF may display specific accessibility distributions that are coordinated with TF expression across the cells, representing cell states in which the TF plays defining regulatory roles. Given *N* metacells, *M* expressed TFs with known motifs, and *L* peaks, we define a peak accessibility matrix $X_{1}=R^{L\times N}$, in which each entry represents peak accessibility in the corresponding metacell. Instead of examining peak accessibility in each metacell, which is inherently noisy, we use singular value decomposition (SVD) (Klema and Laub 1980) of the peak accessibility matrix to learn a low-dimensional embedding of the peaks by estimating the latent components that may be informed by cell states (Fig. 1c).

(2) *Sequence feature representation.* We also utilize sequence features encoding TF binding motif patterns that may convey the binding of a given TF as well as its potential co-binding partners. Applying motif scanning, we define a binary peak-motif matrix $X_{2}\in R^{L\times M}$ that represents whether a TF motif is detected in a peak. Rather than treating each TF independently (as is common practice), we perform SVD on the peak-motif matrix to infer latent components that may capture TF motif presence in peaks and learn a low-dimensional feature embedding of the peaks (Fig. 1d). Specifically, for TF partners in the same regulatory modules with co-binding behaviors, or TFs sharing similar motifs, binding motifs may co-occur across multiple peaks and are likely to share latent component affiliations, such that motifs or motif variants that are missed by motif scanning may still be recovered through these co-occurrence patterns. We choose *d*_1_ = 50 and *d*_2_ = 50 chromatin accessibility and sequence features, respectively (see Supplementary Material and Supplementary Figs S1 and S2 for discussions on feature dimensions). The two resulting latent spaces provide discriminatory features for a logistic regression classifier, and also help guide the selection of pseudo-labeled positive and negative training examples.

#### 3.2.2 Peak clustering in paired feature spaces and peak enrichment analysis

Each peak is thus placed on two manifolds: one in chromatin accessibility and the other in sequence feature space, based on the embeddings above. Next, on each manifold, we cluster similar peaks using PhenoGraph (Levine *et al.* 2015), such that each peak is assigned a pair of cluster labels, one for each manifold (Fig. 1e). The key assumption of Rediscover is that peaks sharing the same cluster label on both manifolds are also likely to share TF binding class labels; thus, for each TF, we use these paired cluster labels to help define positive and negative training samples for our classifier. Let $C_{k}^{\left(\text{ATAC}\right)},\;C_{l}^{\left(\text{seq}\right)}$ represent the peak group *k* in the accessibility feature space, and group *l* in the sequence feature space, respectively. Let *S_kl_* denote the subset of peaks with the paired group assignment (*k*, *l*). We have $S_{kl}=C_{k}^{\left(\text{ATAC}\right)}\cap C_{l}^{\left(\text{seq}\right)}$.

Let $V_{\text{motif}}^{\left(j\right)},\;{\bar{V}}_{\text{motif}}^{\left(j\right)}$ denote the sets of peaks with or without the motif of TF *j* detected by motif scanning, respectively. Let $V_{\text{Unify}}^{\left(j\right)},\;{\bar{V}}_{\text{Unify}}^{\left(j\right)}$ denote the sets of peaks predicted or not predicted to be bound by TF *j* by Unify, respectively. We have $V_{\text{Unify}}^{\left(j\right)}\subset V_{\text{motif}}^{\left(j\right)}$. For each TF, to help select positive training samples, we perform enrichment analysis of the peaks with predicted TF binding in the different paired groups (Fig. 1f). Suppose there are *a_kl_* peaks in total and *b_kl_* peaks from $V_{\text{Unify}}^{\left(j\right)}$ in *S_kl_*, respectively. For each *S_kl_*, we merge all the other peaks as the alternative group, and perform Fisher’s exact test to estimate the statistical significance that the peaks from $V_{\text{Unify}}^{\left(j\right)}$ are enriched in *S_kl_*. We assume peaks from the paired group with significant enrichment of peaks in $V_{\text{Unify}}^{\left(j\right)}$ are more likely to be bound by the corresponding TF.

It is important to select good negative training samples that are unlikely to be bound by the TF. Since we assume that there can be TF-bound peaks without an identified motif, we use similarity in the latent space to help perform initial TFBS prediction for the peaks without the TF motif detected. To ensure that fewer true positives are selected for the negative training set, we define candidate peaks in a permissive manner. If *S_kl_* contains peaks from $V_{\text{Unify}}^{\left(j\right)}$ ($S_{kl}\cap V_{\text{Unify}}^{\left(j\right)}\neq \emptyset$), we include all peaks in *S_kl_* ($S_{kl}\cap {\bar{V}}_{\text{motif}}^{\left(j\right)}$) as the new candidate peaks with potential binding of TF *j*. We denote the approach as Unify + feature group. Next, we look for the neighbors of each peak from $V_{\text{Unify}}^{\left(j\right)}$ on the two manifolds. Let $Nbr_{K}^{\left(\text{ATAC}\right)}(i),\;Nbr_{K}^{\left(\text{seq}\right)}(i)$ represent the *K* nearest neighbors of peak *i* in the accessibility and sequence feature spaces, respectively (e.g. *K *=* *100). Let

$$\begin{array}{c} Nbr_{K}^{\left(\text{ATAC},j\right)}=\underset{i\in V_{\text{Unify}}^{\left(j\right)}}{\cup }\{Nbr_{K}^{\left(\text{ATAC}\right)}(i)\},\\ Nbr_{K}^{\left(\text{seq},j\right)}=\underset{i\in V_{\text{Unify}}^{\left(j\right)}}{\cup }\{Nbr_{K}^{\left(\text{seq}\right)}(i)\}. \end{array}$$

Suppose $Nbr_{K}^{\left(j\right)}=Nbr_{K}^{\left(\text{ATAC},j\right)}\cup Nbr_{K}^{\left(\text{seq},j\right)}$, representing the union of *K* nearest neighbors of peaks in $V_{\text{Unify}}^{\left(j\right)}$ on the two manifolds, which have considerable probability of TF binding.

#### 3.2.3 Pseudo-labeled sample selection and model training

Next, we perform semi-supervised learning utilizing selected pseudo-labeled training samples (i.e. pseudo semi-supervised learning) to train a prediction model that can predict TF binding in each peak locus for the given TF (Fig. 1g). Here, we want to train a classifier that can capture the representative sequence and chromatin accessibility patterns of peaks with TF motifs and with high Unify-estimated TF binding score, and use it to predict TF binding in peaks without motifs.

(1) *Pseudo labeled training sample selection*

(1.1) *Selection of pseudo positive training sample*. For a given TF, the pseudo positive samples are selected from peaks with TF binding predicted by Unify. We wish to build a representative positive training set, cognizant of potential false positives from Unify. We assume higher scoring peaks are more likely true positives. Therefore, for each peak predicted to be bound by TF *j*, we normalize the peak accessibility-TF expression correlation (denoted as $\rho_{\mathrm{peak},TF}$) and the estimated TF binding score (score 1 by Unify) between 0 and 1 by quantile transformation, respectively. Suppose the two normalized scores are $s_{i,1}^{\left(j\right)}$ and $s_{i,2}^{\left(j\right)}$ for peak *i* and TF *j*. We also assume peak enrichment in the paired group assignment more likely indicates true positive. Suppose peak *i* has paired group assignment (*k*, *l*) (peak $i\in S_{kl}$). If *S_kl_* is significantly enriched for peaks with predicted TF binding (peaks in $V_{\text{Unify}}^{\left(j\right)}$), we use lower thresholds on $s_{i,1}^{\left(j\right)}$ and $s_{i,2}^{\left(j\right)}$ to select peak *i* as a pseudo positive sample ($s_{i,1}^{\left(j\right)}>\tau_{1}$ or $s_{i,2}^{\left(j\right)}>\tau_{1}$). If *S_kl_* is not enriched for peaks from $V_{\text{Unify}}^{\left(j\right)}$, we use higher thresholds for selection ($s_{i,1}^{\left(j\right)}>\tau_{2}$ or $s_{i,2}^{\left(j\right)}>\tau_{2},\;\tau_{2}>\tau_{1}$). On the data in this work, we used $\tau_{1}=0.25,\;\tau_{2}=0.75$.

(1.2) *Selection of pseudo negative training sample.* The pseudo negative samples are selected from two groups of peaks: (i) peaks with the motif detected but without TF binding predicted by Unify ($V_{\text{motif}}^{\left(j\right)}\cap {\bar{V}}_{\text{Unify}}^{\left(j\right)}$), and (ii) peaks without the motif detected (${\bar{V}}_{\text{motif}}^{\left(j\right)}$). To ensure true negatives, for (i) and (ii), we select the pseudo negative samples mainly with three criteria: (a) no predicted TF binding by the Unify + feature group method as described above, (b) not in a paired group significantly enriched with peaks from $V_{\text{Unify}}^{\left(j\right)}$, and (c) not from the peak neighbor set $Nbr_{K}^{\left(j\right)}$ as defined above. Suppose we select *n*_1_ pseudo positive peaks in (1.1), we select $n_{2,1}=r_{1}n_{1}$ and $n_{2,2}=r_{2}n_{1}$ pseudo negative peaks from (i) and (ii), respectively. We choose $r_{1}=0.25,\;r_{2}=1.5$ as we assume that there are more negative peaks and we do not induce high class imbalance in the selected samples.

(2) *Training a TF binding prediction model.* For each peak, we concatenate the *d*_1_-dimensional chromatin accessibility feature embedding and the *d*_2_-dimensional sequence feature embedding as the feature vector of the corresponding peak. We then train a classification model using the selected pseudo-labeled samples and apply the trained model to predict TF binding in all the peak loci. We specifically use the logistic regression model as the classifier, which provides better prediction performance than the XGBoost classifier (Chen and Guestrin 2016) based on the evaluation (Supplementary Fig. S3).

## 4 Results

### 4.1 Evaluation of TF binding prediction performance

We applied REUNION to predict TF binding and infer peak-TF-gene regulatory associations in single-cell multiome data from human peripheral blood mononuclear cells (PBMCs) (10x Genomics) that were pre-processed in Persad *et al.* (2023). We applied the SEACells algorithm (Persad *et al.* 2023) to estimate 490 metacells from the scRNA-seq data, generating aggregated gene expression and peak accessibility read counts that are normalized per metacell and log-transformed. To benchmark performance, we used 67 public ChIP-seq datasets from databases including ENCODE (Luo *et al.* 2020) and Cistrome DB (Zheng *et al.* 2019, Mei *et al.* 2016), consisting of unique combinations of 59 TFs and four cell types (B cell, T cell, monocyte and macrophage) (Supplementary Material). Each ATAC-seq peak that overlaps with a ChIP-seq peak was labeled as positive for the corresponding TF, and was otherwise labeled as negative.

#### 4.1.1 Evaluation of genome-wide TF binding predictions

We evaluated the performance of REUNION on TF binding prediction in genome-wide peaks (with or without detected motifs), compared to three types of methods: (i) TFBS prediction or GRN inference utilizing single-cell multiome data, including the representative methods SCENIC+ (Bravo González-Blas *et al.* 2023), *in silico* ChIP-seq (Argelaguet et al. 2022), GRaNIE (Kamal *et al.* 2023), Pando (Fleck *et al.* 2023), and TRIPOD (Jiang *et al.* 2022); (ii) TF motif scanning of DNA sequences alone, utilizing different motif collections; and (iii) TF footprinting analysis-based methods, as exemplified by TOBIAS (Bentsen *et al.* 2020).

Methods in categories (i) and (iii) all require motif scanning results as input. For category (ii), we directly compared with TF motif detection results utilizing the curated CIS-BP motif collection (Weirauch *et al.* 2014), HOCOMOCO motifs (with PWMScan for motif scanning) (Ambrosini *et al.* 2018, Kulakovskiy *et al.* 2018), Pando motif collection (Fleck *et al.* 2023), and JASPAR motifs (Fornes *et al.* 2020), which were used by *in silico* ChIP-seq, GRaNIE, Pando, and TRIPOD, respectively. REUNION and TOBIAS also used CIS-BP motifs. SCENIC+ uses the pycistarget package to perform TFBS prediction by motif enrichment analysis, utilizing a large curated motif database which integrates many existing motif collections (see Supplementary Material for details on how the methods were run).

For methods that generate continuous scores measuring the association strength between a peak and the paired TF, we used AUPR (area under the precision-recall curve) and precision at 15% or 20% recall to evaluate TFBS prediction performance, as the continuous score allows us to derive peak-TF associations at different thresholds. For REUNION, the association scores are the TF binding probabilities predicted by Rediscover; for TOBIAS and *in silico* ChIP-seq, they are TF binding scores; for other multiome-based GRN inference methods, they are adjusted *P*-values measuring the significance of estimated peak-TF or peak-TF-gene connections; and for motif scanning using the CIS-BP motif collection, they are the reported motif scores (Supplementary Material).

Methods that do not generate continuous scores are not amenable to AUPR, so we evaluated these with the *F*_1_ score, computed by comparing the predicted peak-TF links with the TF binding events supported by ChIP-seq data for each analyzed TF. For motif scanning with collections other than CIS-BP, binary motif detection results were provided by the multiome-based method that utilized the collection, with scanning performed as an internal step or based on a pre-built TFBS database. SCENIC+ predicts TFBSs using motif enrichment analysis, and outputs binary peak-TF link predictions without association scores.

**Figure 2.**
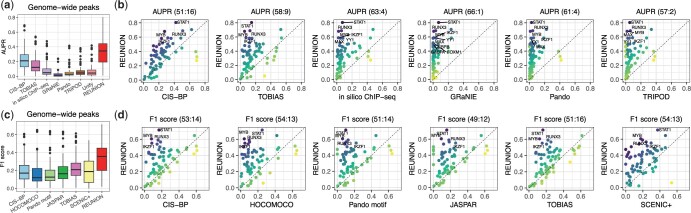
Performance of TF binding prediction in genome-wide peaks with or without detected TF motifs, based on ChIP-seq data for 59 TFs in human PBMC data. (a) AUPR distributions of TFBS predictions for the evaluated TF ChIP-seq datasets by different methods. (b) Pairwise comparisons of REUNION with individual methods, plotting TFBS predictions for each ChIP-seq dataset. (c) *F*_1_ score distributions of TFBS predictions by different methods. (d) Pairwise *F*_1_ score comparisons between REUNION and motif scanning using different motif collections, TOBIAS, and SCENIC+. In (b, d), each dot represents a TF ChIP-seq dataset, with color scaled by the performance difference between REUNION and the compared method. The number of TF ChIP-seq datasets for which REUNION has higher (*n*_1_) or lower (*n*_2_) performance than the method under comparison by at least 0.001 appears above each plot as ($n_{1}:n_{2}$). The similar notation applies to Fig. 3.

**Figure 3.**
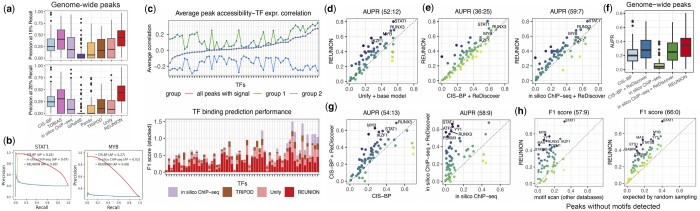
Additional performance analysis of TF binding prediction in genome-wide peaks with or without detected TF motifs in human PBMC data. (a) Precision at 15% (top) or 20% (bottom) recall of TFBS predictions. (b) Precision–recall curves of TFBS predictions for STAT1 and MYB by REUNION compared with *in silico* ChIP-seq and motif scanning using the CIS-BP motif collection. AP represents AUPR. (c) Top: Average $\rho_{\mathrm{peak},TF}$ across all ATAC-seq peaks with ChIP-seq signals for each TF (all peaks), ranked from lowest to highest, or across the subset of peaks without repression ($\rho_{\mathrm{peak},TF}>-0.05$, group 1) or with potential repression ($\rho_{\mathrm{peak},TF}<-0.05$, group 2). Bottom: Stacked *F*_1_ scores for predictions by four methods. Each bar corresponds to one TF, ordered as in the top plot. (d, e) TFBS prediction performance of REUNION compared to Rediscover with (d) no clustering-guided pseudo training sample selections or with (e) motif scanning results (left) or *in silico* ChIP-seq predictions (right) as input. (f) AUPR of genome-wide TFBS predictions summarized across all TFs for multiple methods. (g) TFBS prediction performance of motif scanning with the CIS-BP motif collection compared with using motif-scanning predictions as input to Rediscover (left), and performance of *in silico* ChIP-seq compared with using *in silico* ChIP-seq predictions as input to Rediscover (right). (h) Prediction performance of REUNION in peaks without the TF motif detected compared with motif scanning predictions using other motif databases (left) or compared with average scores based on random sampling (right).

We found that REUNION achieves relatively high genome-wide TF binding prediction performance on the PBMC data (median AUPR = 0.34 and median *F*_1_ score = 0.36 across ChIP-seq datasets), and outperforms the other tested methods (Figs 2 and 3a, Supplementary Figs S4 and S5) on average. REUNION predictions attain substantially higher AUPR than other single-cell multiome methods, and higher *F*_1_ scores than the leading GRN inference method SCENIC+ as well as motif scanning-based predictions using each of the four comprehensive motif collections. Its predictions outperform predictions using CIS-BP motifs by AUPR (where motif scores are available to compute AUPR) and by *F*_1_ score. Moreover, REUNION outperforms the top-ranking TF footprinting-based method TOBIAS by AUPR, *F*_1_ score, and precision at 20% recall on average, though the methods have similar precision at 15% recall (Figs 2 and 3a). Notably, TOBIAS requires deeply sequenced bulk ATAC-seq data, and therefore cannot be directly applied to single-cell data to predict binding. At the level of individual TFs, we found that REUNION is consistently better at capturing the corresponding TFBSs of multiple TFs with important functions in PBMCs, such as STAT1, IKZF1 and MYB (Fig. 2b and d). The precision-recall curves for individual TFs such as STAT1 and MYB (Fig. 3b) show higher performance over varying thresholds.

To assess the ability of REUNION to predict the binding of TFs with different chromatin accessibility-TF expression covariation patterns, we sorted TFs by the average correlation between peak accessibility and TF expression (noted as average $\rho_{\mathrm{peak},TF}$) across all the ATAC-seq peaks with ChIP-seq signals for the corresponding TF (Fig. 3c). We found that REUNION performs better on average than alternative methods, even when TFs are separated into either strong or relatively weak average $\rho_{\mathrm{peak},TF}$ groups.

We also found that REUNION outperforms a model variant in which we omitted the clustering-guided pseudo-labeled training sample selection steps in Rediscover (noted as “base model”), supporting the importance of selecting reliable training samples (Fig. 3d). To determine how informative Unify predictions are to Rediscover, we assessed alternative inputs. Specifically, we used TFBS predictions by motif scanning with the CIS-BP motif collection and the *in silico* ChIP-seq method as input to Rediscover for TFBS prediction (Fig. 3e). REUNION performed better than the two alternatives on average, emphasizing the contribution provided by Unify (Fig. 3e and f). Our results demonstrate that Rediscover is flexible with respect to input, and can be used to substantially boost the performance of other methods, such as motif scanning or *in silico* ChIP-seq, compared to running them alone (Fig. 3f and g). We further evaluated REUNION when restricting the analysis to peaks harboring detected motifs, and found that it maintains higher average *F*_1_ scores across the ChIP-seq datasets than multiome-based GRN inference methods (Supplementary Fig. S6). REUNION predictions in peaks with detected motifs are based on a refinement of Unify estimates by Rediscover; thus, Rediscover enhances the performance of Unify, enabling REUNION to gain more advantage over the other methods.

#### 4.1.2 TFBS prediction in peaks without the TF motif detected

We evaluated the TF binding prediction performance of REUNION in peaks without the TF motif detected on the PBMC data, as this is an important innovation of the method. Our benchmarking in Fig. 2 demonstrates that the REUNION classifier is quite accurate at associating TFs with peaks lacking detected motifs, a key factor behind REUNION’s substantially superior recall. As alternative methods utilizing single-cell multiome data do not consider TF associations to peaks without the detected motifs, we compared REUNION to TFBS prediction based on the union of motif scanning results utilizing other motif collections (Fig. 3h). Based on *F*_1_ scores, REUNION outperforms this approach on average, implying that the peaks bound by the TF but without the motif detected may encode underexplored sequence patterns that are not well represented in motif databases. Furthermore, we performed Fisher’s exact test and the chi-squared test to examine if the TFBSs predicted by Rediscover are enriched in the peaks with ChIP-seq signals. Predictions for all the evaluated TFs have *P*-value <1e−03 for both tests except for PKNOX1. Moreover, for a given TF, we randomly shuffled the predicted positive labels among peaks without the detected motif (*n *=* *100 times) to generate random predictions, and calculated an *F*_1_ score for each set of random predictions to form a background distribution, using the mean value as the expected *F*_1_ score (Supplementary Fig. S7). REUNION exceeds the expected performance on all the ChIP-seq datasets (Fig. 3h). Our observations demonstrate that REUNION can effectively identify TF binding activities beyond known TF motifs.

#### 4.1.3 Sequence and motif enrichment analysis of peaks without detected TF motifs

**Figure 4.**
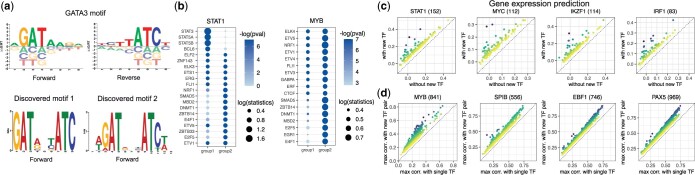
REUNION recovers true peak-TF associations in accessibility peaks for which motif scanning fails to detect motifs. (a) GATA3 motif in the CIS-BP collection (top) and potential GATA3 motifs discovered by STREME in peaks for which motif scanning failed to detect GATA3 motifs but TF binding was evidenced by ChIP-seq signals and predicted by Rediscover (bottom). (b) Motif enrichment analysis for STAT1 and MYB, using Fisher’s exact test for two types of peaks overlapping with TF ChIP-seq signals: peaks with detected TF motif (group 1) and peaks without the detected motif but with TF binding correctly predicted by Rediscover (group 2). Each row corresponds to a TF with top ranking significant motif enrichment in either group 1 or group 2 peaks for the given query TF. Color and size of each dot correspond to $-{\;\text{log}\;}_{10}(P-\text{value})$ and ${\;\text{log}\;}_{10}(\text{statistics value})$, respectively. (c) Adding new gene-TF associations, based on predicted TF binding in peaks without a detected motif of the given TF, improves gene expression predictions (measured by Pearson correlation between predicted and observed gene expressions after adding the association). Each dot in these four examples corresponds to a potential new target gene of the TF. (d) Maximal correlation between peak accessibility and the product of the expression of (i) one existing linked TF predicted by Unify and (ii) one new linked TF predicted by Rediscover, is higher than the maximal correlation between peak accessibility and the single associated TF expression (including the existing and new linked TFs). For (c, d), the new linked TF and the number of genes (c) or peaks (d) satisfying the selection criteria are shown in the parentheses above the plot.

Motif scanning may fail to identify a true TFBS in a peak for at least two reasons. The first is that motif collections may miss noncanonical motif variations, such as sites bound by a dimer rather than a monomer, or other variations that appear less often than a dominant motif and score lower during motif scanning (Lambert *et al.* 2018). We used STREME (Bailey 2021) for *ab initio* sequence-based motif discovery in the set of peaks that lack detected motifs but harbor ChIP-seq signals and REUNION-predicted binding for a given TF (we refer to these as “Group 2 peaks”), and found potential noncanonical motifs. For example, STREME identified a putative dimer motif for GATA3 that is absent from the utilized CIS-BP collection, and also deviates from the known GATA3 dimer motifs found in the existing motif collections (Fig. 4a, Supplementary Fig. S8).

The second reason is that a TF may depend on cofactor(s) to various extents or in different contexts for binding, and thus provide weak or no binding signals on its own. We performed enrichment analysis for motifs of the other TFs in Group 2 peaks of a given TF, and recovered significantly enriched motifs (examples are shown in Fig. 4b). Specifically, for STAT1, we identified enriched motifs of NRF1, SMAD5, DNMT1, MBD2, and E4F1 in Group 2 peaks. Of these, NRF1 and E4F1 are known co-regulators of STAT1 (Liu *et al.* 2008). This is in addition to other known co-regulators such as STAT3 and STAT5A, which have motifs enriched in the peaks with detected motifs of STAT1 (“Group 1 peaks”).

Our ability to find enriched motifs of other TFs in Group 2 peaks is likely due to the integrated latent space utilized by Rediscover, which is designed to capture potential modularity in peak-TF behavior. Specifically, co-regulatory TFs may have correlated expression, and target peaks may have correlated accessibility and shared sequence signatures that allow for binding to be inferred even when motifs of the given TF are hidden. Thus, the latent space utilized by Rediscover is able to help improve TF binding prediction.

### 4.2 TF binding prediction in peaks without the detected motif reveals new potential gene-TF associations and TF interactions

We asked whether TF binding prediction in peaks without the detected motif may help discover new gene-TF associations, by linking the TF to the potential target gene through putative peak-gene links. There are two scenarios: (i) the TF is already associated with the target gene via alternative peak-gene links predicted by Unify, or (ii) the TF is not associated with the target gene by Unify, and a new putative gene-TF link is generated. Here, we focus on the second scenario. For each TF with predicted binding in peaks without the detected motif, we link the TF to target genes through the candidate peak-gene links previously identified by Unify prior to applying the two scoring functions. We infer associations between the TF and target gene by predicting gene expression using the TF expression and comparing the prediction performance between utilizing two sets of predictor variables (Supplementary Material). Both sets contain the candidate regulatory TFs of the gene previously estimated by Unify, and the second set also includes the newly linked TF. We found that for many TFs, there may exist new potential target genes for which including the given TF as a predictor improves gene expression prediction accuracy measured by the Pearson correlation between the predicted and real gene expressions by at least 0.05. Examples include STAT1, MYC, IKZF1, IRF1 (Fig. 4c). These observations support the utility of performing TFBS prediction in peaks without the detected motif, which may recover potential missing target genes of the TFs.

Next, we explored whether predicting TF binding in peaks without the detected motif may reveal potential hidden interaction effects of TF partners on chromatin accessibility. For peak *i*, let *T_i_* and ${\hat{T}}_{i}=T_{i}\cup \{\text{TF}\;j\}$ represent the estimated binding TFs before and after applying Rediscover to predict the binding sites of TF *j*, respectively. We compute two types of correlations using the set ${\hat{T}}_{i}$: (i) the correlation between peak accessibility and the expression of each single TF ($\rho_{\mathrm{peak},TF}$), and (ii) the correlation between the peak accessibility and the product of the expression of two TFs in a pair ($\rho_{\mathrm{peak},TF\;\mathrm{pair}}$). Comparing (i) and (ii), we look for the possibilities that (a) the expression product of two paired TFs has higher correlation with the peak accessibility than each single TF linked to the peak; and (b) the TF pair reaching the highest correlation in (i) involves the new predicted binding TF *j*. We find numerous examples of potential TF interaction effects on the peak accessibility (Fig. 4d). In particular, we observed a large number of peaks for which MYB contributes to $\rho_{\mathrm{peak},TF\;\mathrm{pair}}$ higher than $\rho_{\mathrm{peak},TF}$. The observations suggest that predicting TF binding in peaks without the detected motif may help reveal possible co-binding TF partners with interacting regulatory effects on the accessibility of the putative target peaks.

## 5 Discussion

In this study, we developed REUNION, a computational framework composed of Unify and Rediscover to perform genome-wide TF binding prediction and regulatory association inference from single-cell multiome data. Unify leverages joint mutual information-inspired complementary score functions to address the challenge of incorporating TF, peak, and target gene simultaneously to infer peak-TF-gene associations. Rediscover is unique in predicting TF binding in genomic regions without the detected motif, based on pseudo semi-supervised learning on single-cell multiome data (without the need for ChIP-seq data). This approach to peak-TF association recovery is different from existing supervised learning-based TFBS prediction methods, and addresses the challenge of incomplete detection by commonly used motif scanning approaches. On PBMC data, REUNION achieves high average TFBS prediction performance compared to other representative methods, showing its potential for more comprehensive discovery of TF binding activities beyond motif scanning results.

There are several directions for the improvement of REUNION. First, Unify computes scores for individual peak-TF-gene links, whereas it would be useful to model the potential combinatorial functions between TFs or peaks. Second, the performance of Rediscover critically depends on the feature representations of the peaks and the quality of the selected pseudo-labeled training samples, such that we could integrate more feature types to enhance the feature embeddings of the peaks. For example, we could use *k*-mer frequency to form the feature representation. Third, the pseudo training sample selection is a relatively new task for TFBS prediction, which could benefit from utilizing better defined constraints based on the peak distributions on the feature manifolds and from improving the association predictions by Unify. Finally, Rediscover currently trains TF-specific models for each TF. There may exist combinatorial effects of TFs which involve co-binding of TF partners or competitions between TFs. It would be more effective to learn prediction models jointly for TF combinations.

We plan to perform more extensive analyses of potential TF partners identified in REUNION-predicted peak-TF links, including utilizing protein-protein interaction databases for evaluation (Szklarczyk *et al.* 2021). We will also seek more performance evaluation approaches that take potential false positive ChIP-seq signals into account. Furthermore, it will be interesting to investigate the sequence elements predicted by Rediscover to participate in TF binding, without detected binding motifs. Recovering the potential hidden motifs or TF co-binding patterns from these sequences would advance our knowledge of TF binding behavior and facilitate the study of gene regulatory mechanisms.

## 6 Conclusion

REUNION provides a computational framework for genome-wide TF binding prediction and regulatory association inference from single-cell multi-omics data. REUNION may help reveal underexplored regulatory associations and has the potential to advance the study of gene regulation mechanisms.

## Supplementary Material

btae234_Supplementary_Data

## Data Availability

The publicly available 10x Genomics PBMC multiome data used in this study were sourced from https://www.10xgenomics.com/resources/datasets/pbmc-from-a-healthy-donor-granulocytes-removed-through-cell-sorting-10-k-1-standard-2-0-0.

## References

[btae234-B1] Ambrosini G , Groux R, Bucher P. PWMScan: a fast tool for scanning entire genomes with a position-specific weight matrix. Bioinformatics 2018;34:2483–4. 29514181 10.1093/bioinformatics/bty127 PMC6041753

[btae234-B2] Argelaguet R , Lohoff T, Li JG et al Decoding gene regulation in the mouse embryo using single-cell multi-omics. bioRxiv, 2022, preprint: not peer reviewed. 10.1101/2022.06.15.496239.

[btae234-B3] Bailey TL. STREME: accurate and versatile sequence motif discovery. Bioinformatics 2021;37:2834–40. 33760053 10.1093/bioinformatics/btab203 PMC8479671

[btae234-B4] Bennasar M , Hicks Y, Setchi R. Feature selection using joint mutual information maximisation. Expert Syst Appl 2015;42:8520–32.

[btae234-B5] Bentsen M , Goymann P, Schultheis H et al ATAC-seq footprinting unravels kinetics of transcription factor binding during zygotic genome activation. Nat Commun 2020;11:4267. 32848148 10.1038/s41467-020-18035-1 PMC7449963

[btae234-B6] Bravo González-Blas C , De Winter S, Hulselmans G et al SCENIC+: single-cell multiomic inference of enhancers and gene regulatory networks. Nat Methods 2023;20:1355–67. 37443338 10.1038/s41592-023-01938-4 PMC10482700

[btae234-B7] Cao J , Cusanovich DA, Ramani V et al Joint profiling of chromatin accessibility and gene expression in thousands of single cells. Science 2018;361:1380–5. 30166440 10.1126/science.aau0730 PMC6571013

[btae234-B8] Cazares TA , Rizvi FW, Iyer B et al maxATAC: Genome-scale transcription-factor binding prediction from ATAC-seq with deep neural networks. PLoS Comput Biol 2023;19:e1010863. 36719906 10.1371/journal.pcbi.1010863 PMC9917285

[btae234-B9] Chen C , Hou J, Shi X et al DeepGRN: prediction of transcription factor binding site across cell-types using attention-based deep neural networks. BMC Bioinformatics 2021;22:1–18. 33522898 10.1186/s12859-020-03952-1 PMC7852092

[btae234-B10] Chen S , Lake BB, Zhang K. High-throughput sequencing of the transcriptome and chromatin accessibility in the same cell. Nat Biotechnol 2019;37:1452–7. 31611697 10.1038/s41587-019-0290-0 PMC6893138

[btae234-B11] Chen T , Guestrin C. Xgboost: A scalable tree boosting system. In: *Proceedings of the 22nd ACM SIGKDD International Conference on Knowledge Discovery and Data Mining* 2016;785-794.

[btae234-B12] Fleck JS , Jansen SMJ, Wollny D et al Inferring and perturbing cell fate regulomes in human brain organoids. Nature 2023;621:365–72. 36198796 10.1038/s41586-022-05279-8 PMC10499607

[btae234-B13] Fornes O , Castro-Mondragon JA, Khan A et al JASPAR 2020: update of the open-access database of transcription factor binding profiles. Nucleic Acids Res 2020;48:D87–92. 31701148 10.1093/nar/gkz1001 PMC7145627

[btae234-B14] Fu L , Zhang L, Dollinger E et al Predicting transcription factor binding in single cells through deep learning. Sci Adv 2020;6:eaba9031. 33355120 10.1126/sciadv.aba9031 PMC11206197

[btae234-B15] Jiang Y , Harigaya Y, Zhang Z et al Nonparametric single-cell multiomic characterization of trio relationships between transcription factors, target genes, and cis-regulatory regions. Cell Syst 2022;13:737–51.e4. 36055233 10.1016/j.cels.2022.08.004 PMC9509445

[btae234-B16] Kamal A , Arnold C, Claringbould A et al GRaNIE and GRaNPA: inference and evaluation of enhancer-mediated gene regulatory networks. Mol Syst Biol 2023;19:e11627.37073532

[btae234-B17] Kartha VK , Duarte FM, Hu Y et al Functional inference of gene regulation using single-cell multi-omics. Cell Genom 2022;2:100166. 36204155 10.1016/j.xgen.2022.100166 PMC9534481

[btae234-B18] Keilwagen J , Posch S, Grau J. Accurate prediction of cell type-specific transcription factor binding. Genome Biol 2019;20:9–17. 30630522 10.1186/s13059-018-1614-y PMC6327544

[btae234-B19] Klema V , Laub A. The singular value decomposition: its computation and some applications. IEEE Trans Automat Contr 1980;25:164–76.

[btae234-B20] Kulakovskiy IV , Vorontsov IE, Yevshin IS et al HOCOMOCO: towards a complete collection of transcription factor binding models for human and mouse via large-scale ChIP-Seq analysis. Nucleic Acids Res 2018;46:D252–9. 29140464 10.1093/nar/gkx1106 PMC5753240

[btae234-B21] Lambert SA , Jolma A, Campitelli LF et al The human transcription factors. Cell 2018;172:650–65. 29425488 10.1016/j.cell.2018.01.029 PMC12908702

[btae234-B22] Levine JH , Simonds EF, Bendall SC et al Data-driven phenotypic dissection of AML reveals progenitor-like cells that correlate with prognosis. Cell 2015;162:184–97. 26095251 10.1016/j.cell.2015.05.047 PMC4508757

[btae234-B23] Li H , Quang D, Guan Y. Anchor: trans-cell type prediction of transcription factor binding sites. Genome Res2019a;29:281–92. 30567711 10.1101/gr.237156.118 PMC6360811

[btae234-B24] Li Z , Schulz MH, Look T et al Identification of transcription factor binding sites using ATAC-seq. Genome Biol2019b;20:45. 30808370 10.1186/s13059-019-1642-2 PMC6391789

[btae234-B25] Liu X , Yu X, Zack DJ et al TiGER: a database for tissue-specific gene expression and regulation. BMC Bioinformatics 2008;9:271. 18541026 10.1186/1471-2105-9-271 PMC2438328

[btae234-B26] Luo Y , Hitz BC, Gabdank I et al New developments on the Encyclopedia of DNA Elements (ENCODE) data portal. Nucleic Acids Res 2020;48:D882–9. 31713622 10.1093/nar/gkz1062 PMC7061942

[btae234-B27] Ma S , Zhang B, LaFave LM et al Chromatin potential identified by shared single-cell profiling of RNA and chromatin. Cell 2020;183:1103–16.e20. 33098772 10.1016/j.cell.2020.09.056 PMC7669735

[btae234-B28] Mei S , Qin Q, Wu Q et al Cistrome Data Browser: a data portal for ChIP-Seq and chromatin accessibility data in human and mouse. Nucleic Acids Res 2016;45:D658–62. 27789702 10.1093/nar/gkw983 PMC5210658

[btae234-B29] Persad S , Choo Z-N, Dien C et al SEACells infers transcriptional and epigenomic cellular states from single-cell genomics data. Nat Biotechnol 2023;41(12):1746–57. 36973557 10.1038/s41587-023-01716-9 PMC10713451

[btae234-B30] Quang D , Xie X. FactorNet: a deep learning framework for predicting cell type specific transcription factor binding from nucleotide-resolution sequential data. Methods 2019;166:40–7. 30922998 10.1016/j.ymeth.2019.03.020 PMC6708499

[btae234-B31] Schep AN. *motifmatchr: Fast Motif Matching in R*. 2023. 10.18129/B9.bioc.motifmatchr(1 April 2024, date last accessed).

[btae234-B32] Schep AN , Wu B, Buenrostro JD et al chromVAR: inferring transcription-factor-associated accessibility from single-cell epigenomic data. Nat Methods 2017;14:975–8. 28825706 10.1038/nmeth.4401 PMC5623146

[btae234-B33] Stuart T , Srivastava A, Madad S et al Single-cell chromatin state analysis with Signac. Nat Methods 2021;18:1333–41. 34725479 10.1038/s41592-021-01282-5 PMC9255697

[btae234-B34] Szklarczyk D , Gable AL, Nastou KC et al The STRING database in 2021: customizable protein–protein networks, and functional characterization of user-uploaded gene/measurement sets. Nucleic Acids Res 2021;49:D605–12. 33237311 10.1093/nar/gkaa1074 PMC7779004

[btae234-B35] Wang Y , Yuan P, Yan Z et al Single-cell multiomics sequencing reveals the functional regulatory landscape of early embryos. Nat Commun 2021;12:1247. 33623021 10.1038/s41467-021-21409-8 PMC7902657

[btae234-B36] Weirauch MT , Yang A, Albu M et al Determination and inference of eukaryotic transcription factor sequence specificity. Cell 2014;158:1431–43. 25215497 10.1016/j.cell.2014.08.009 PMC4163041

[btae234-B37] Zheng R , Wan C, Mei S et al Cistrome Data Browser: expanded datasets and new tools for gene regulatory analysis. Nucleic Acids Res 2019;47:D729–35. 30462313 10.1093/nar/gky1094 PMC6324081

