# Supplementary material for: REUNION: transcription factor binding prediction and regulatory association inference from single-cell multi-omics data
Source: Bioinformatics. 2024 Jun 28;40(Suppl 1):i567–75. doi: 10.1093/bioinformatics/btae234 (PMC11211829; doi:10.1093/bioinformatics/btae234)
Supplement: btae234_Supplementary_Data [file btae234_supplementary_data.zip › btae234_Supplementary_Data/Pe'er.249.sup.1.pdf]

REUNION: Transcription factor binding prediction and regulatory association inference from single-cell multi-omics data

(Supplementary Material)

A Supplementary Notes

A.1 Hybrid search strategy for peak-TF-gene association initialization

We design a peak-gene assignment module in Unify to prepare the candidate peak-TF-gene links for using the proposed score function 1 and score function 2. The module comprises the uniquely designed hybrid search for initializing peak-gene links and reduction of association ambiguity through comparison among alternative peak-gene links. The existing methods commonly use gene-based assignment by selecting candidate regulatory peaks within a limited distance of the target gene (typically 50Kb to 250Kb). Longer distance enables the identification of distal regulatory peaks, but also increase the probability of false positive links where the peak is actually connected with other genes and may raise computation cost for GRN inference. Our method combines flexible gene-based search with peak-based search to explicitly specify the alternative peak-gene links in long-distance ranges, accompanied by filtering the possible links through comparisons based on both distance and peak-gene association strength, which facilitates detecting potential long-range regulatory interactions.

Specifically, given a target gene set, we search for peaks within +/-2Mb (or user-defined distance) of the transcription start site (TSS) of each target gene, selecting the candidate regulatory peaks based on the significance of peak accessibility-gene expression correlations (denoted by $\rho_{peak,gene}$) for which the empirical p-values are computed using background peaks matched for average chromatin accessibility and GC content, with uplifted thresholds for longer distances. Next, for a candidate peak identified from above, we seek alternative potential target genes within +/-2Mb of the peak and compute the correlation $\rho_{peak,gene}$. Furthermore, we group the peak-gene links into distance bins and correlation bins. We use the links with the highest correlation in the smallest distance bin and with the smallest distance in the highest correlation bin as the prioritized links to compare against and filter other links, to prepare peak-gene assignments of lower ambiguity and reduce the computation burden of calculating the proposed scores. We then perform TF motif scanning in the candidate peak loci to initially associate the TFs to the peaks and establish the candidate peak-TF-gene links.

A.2 Complementary score functions to infer peak-TF-gene regulatory associations

Given a set of candidate peak-TF-gene links, we design two complementary score functions to jointly measure the association strength of each link and predict the regulatory associations, which are Score function 1: joint score of TF and peak-gene pair, and Score function 2: joint score of peak-TF pair and the potential target gene.

(1) Score function 1 $f(TF; (peak, gene))$ estimates the shared information between the TF and the peak-gene pair, aiming at predicting TF binding activity by not only using the peak-TF correlation, but also incorporating information from the potential target gene of the peak. Suppose X_j, C_i, Y_g represent the expression, accessibility, and expression of TF j, peak i (with the motif of TF j), and the putative target gene g, respectively. Score function 1 is conceptually inspired by the joint mutual

information (JMI) between the TF expression and the combination of the peak accessibility and the target gene expression, which is $I(X_j; C_i, Y_g) = I(X_j, C_i) + I(X_j, Y_g|C_i)$. In practice, estimating the JMI score for continuous variables from the limited data with noise and sparsity is challenging. We choose to use the peak accessibility-TF expression correlation (ρ_{X_j,C_i}) and the partial correlation between target gene and TF expressions conditioned on the peak accessibility ($\rho_{X_j,Y_g;C_i}$) as alternatives to the mutual information and conditional mutual information scores that constitute the JMI score, respectively.

Partial correlation quantifies the strength and direction of the association between two random variables, with the effect of a given set of observed other variables removed. We use Spearman's rank correlation or partial correlation coefficients for the score calculation, which can capture the non-linear relationships between the random variables.

Furthermore, by modifying the normalization approach in the *in silico* ChIP-seq method [1], we estimate a regularization score for each peak-TF pair using the motif scores from motif scanning, which quantifies the confidence level of identifying a TF binding motif in the given DNA sequence, and the peak accessibility. Suppose TF j has motif detected in peak locus i. Suppose the regularization score is $h(\theta_{ij}, c_i)$ for peak i and TF j. We have $h(\theta_{ij}, c_i) \in [0, 1]$, where θ_{ij} represent the motif score of TF j in peak locus i. Specifically, we perform log transformation ($x = log(1 + x)$) of original motif scores and use 95% quantile of the transformed motif scores as the upper bound to scale the scores to be in [0,1] with minmax normalization. The logarithmic transformation is used to mitigate the effect from long-tail distribution of motif scores of specific TF motifs. We use the function $f(x) = 1 - \exp(-a_j x)$ to transform the maximal accessibility across the metacells of the peak locus i with the motif of TF j to a value between [0,1]. The parameter a_j is chosen such that peak with the median maximal accessibility among the peaks with motif detected for the given TF has the score $f(x) = 0.90$. The $\min\max(\cdot)$ represents min-max scaling between 0.5 and 1.

Using score function 1, the TF binding score of TF j in peak locus i (with potential target gene g) is estimated as:

$$s_{ig,j} = (\lambda_1 \rho_{X_j,C_i} + \lambda_2 \rho_{X_j,Y_g;C_i}) \min\max(\tilde{\theta}_{ij}\tilde{\pi}_i),$$

where $\tilde{\theta}_{ij}$, $\tilde{\pi}_i$ represent the normalized motif score of TF motif j in peak locus i, and the transformed maximal laccessibility of peak i in the metacells respectively.

(2) Score function 2 $f((peak, TF); gene)$ estimates the shared information between the peak-TF pair and the potential target gene, in order to infer if the gene is a target of the paired peak and TF. This score function is inspired by the JMI between the target gene expression and the combination of the peak accessibility and TF expression, which is $I(Y_g; C_i, X_j) = I(Y_g, C_i) + I(X_j, Y_g|C_i)$. The score is calculated as:

$$s_{ij,g} = (\eta_1 \rho_{Y_g,C_i} + \eta_2 \rho_{X_j,Y_g;C_i}) \min\max(\tilde{\theta}_{ij}\tilde{\pi}_i),$$

which integrates the correlation between peak accessibility and the potential target gene expression (ρ_{Y_g,C_i}), the partial correlation $\rho_{X_j,Y_g;C_i}$, and a regularization term. In score function 1 and 2, the parameters λ_1, λ_2, η_1, η_2 are adjustable weights of the corresponding correlation or partial correlation. We use $\lambda_1, \lambda_2, \eta_1, \eta_2 \in \{0.5, -0.5\}$ depending on the inferred type of association (activation or repression) between TF, peak, and the potential target gene.

Score function 1 assumes the peak-gene link is given and TF binding in uncertain, while score function 2 assumes peak-TF link is given and the target gene is undetermined. The two scores are complementary to each other and jointly estimate the strength of a peak-TF-gene link.

A.3 Comparison of mechanisms between Unify and the other methods

The *in silico* ChIP-seq library method [1] uses correlation between TF expression and peak accessibility to predict TF binding activity, with regularization by the motif scores from motif scanning and maximum peak locus accessibility across metacells. The method may not be suitable for the scenario where the TF expression is not strongly correlated with peak accessibility. For example, a regulatory region may be widely open across different cell types while the TF expression is cell type-specific. Also, the *in silico* ChIP-seq method did not perform peak-gene association inference. Peaks are linked to genes only by distance threshold. Compared with the *in silico* ChIP-seq method, our metohd not only includes the peak accessibility-TF expression correlation term, but also uses the partial correlation between TF and potential target gene expression given the peak accessibility to borrow information from the potential target gene to assist TF binding prediction.

GRaNIE [2] uses the similar approach as the *in silico* ChIP-seq method to infer peak-TF links and share the similar limitation. GRaNIE additionally uses peak accessibility-target gene expression correlation to predict peak-gene links, followed by compiling the two types of links to build GRN. A limitation is that the two steps of inferring peak-TF links and peak-gene links are performed separately, with pairwise information between features utilized in each step. Only peak-TF-gene links with both significant TF-peak correlations and peak-gene correlations are retained.

Pando [3] uses the product of the TF expression and chromatin accessibility of candidate regulatory peaks (with binding motifs of the TFs) to predict target gene expression by fitting linear regression models. There are several potential limitations. (a) Pando has relatively high time complexity $O(NM^2L^2 + M^3L^3)$, where N is the number of metacells, M and L are the upper bounds of the number of candidate regulatory regions for a gene and the number of TFs with binding motifs detected in a regulatory region, respectively). (b) The model may overfit if the candidate peak number or the number of TFs with motifs in a peak are high, which generate high-dimensional input feature with a large number of interaction terms. (c) The product term may not reflect the real interaction effect between TF and peak accessibility, and may not capture the chromatin accessibility repression effect by TFs.

TRIPOD [4] performs two types of statistics tests of conditional associations, each with two levels of tests, to identify the peak-TF-gene associations. TRIPOD has some potential limitations. First, the time complexity is high $((O(N^{2.5}ML)$ or $(O(N^3ML))$, limiting the scalability to larger sample size. N, M and L are described as above. Specifically, TRIPOD performs non-bipartite matching to find pairs of metacells when matching metacells by TF expression or peak accessibility, which can be considered as finding maximum-weight matching in non-bipartite graphs. The complexity for the non-bipartite maximum-weight matching problem is $O(|V|^2|E|)$ by Edmonds' blossom algorithm [5], or $O(|E|\sqrt{|V|})$ using the more complicated algorithm by [6]. $|V|$ and $|E|$ denote the numbers of nodes and edges in the graph, respectively. Here the nodes in the graph correspond to the N metacells and there are $O(N^2)$ edges. Therefore, for a given peak or TF, the best complexity for matching the metacells is $O(N^{2.5})$ or $O(N^3)$. We need to perform the matching for every candidate peak or TF. The high time complexity of matching metacells limits the scalability of TRIPOD to larger numbers of metacells and identifying regulatory peaks in longer distance. However, a smaller number of metacells leads to a smaller number of matched pairs which may not provide a sufficient sample size for the statistical tests. For example, we observed using 500 metacells reaches better performance than using 100 metacells for TRIPOD on the PBMC data.

Our method is different from the method in mechanism. In the TRIPOD method, matching the metacells pairwise can be considered as discretizing the peak accessibility or TF expression for a

given peak or TF in the N metacells into at most $(N/2)$ levels. Our method does not perform the discretization. Also, our method utilizes the shared information between peak accessibility and TF expression, combined with target gene expression, to predict TF binding in the score function 1. TRIPOD does not use the information between TF and peaks to predict TF-peak links. TRIPOD may not work well if TF expression is highly correlated with peak accessibility. In that case, the metacells matched by TF expression (or peak accessibility) may also have similar peak accessibility (or TF expression). The differential peak accessibility (or TF expression) may be low or not informative. Considering the noise in the data, the correlation between the differential target gene expression and differential peak accessibility (or TF expression) may be low, especially if the potential target gene expression is also highly correlated with the peak accessibility or TF expression. This limitation was revealed in the TF binding prediction performance evaluation. We found that TRIPOD has relatively low prediction performance for specific TFs with relatively high average peak accessibility-TF expression correlation across the peaks with the ChIP-seq signals (Fig. 3c).

A.4 Evaluating prediction performance using varied feature dimensions

In the implementation of Rediscover, We choose d_1=50 and d_2=50 chromatin accessibility and sequence features for each peak locus after singular value decomposition (SVD), respectively. We concatenate the accessibility feature vector and the sequence feature vector to form a $(d_1 + d_2)$-dimensional feature vector for each peak locus. Here we experiment with different choices of the number of feature dimensions. We evaluate how the varied feature dimensions influence the TFBS prediction performance.

Specifically, we use different numbers of latent feature dimensions in each feature space (the chromatin accessibility feature space and the sequence feature space) to generate the feature representations of the peaks and use Rediscover for TF binding prediction in genome-wide peaks for each evaluated TF. We change the number of latent accessibility feature dimension d_1 and the number of latent sequence feature dimension d_2 synchronously ($d_1 = d_2$), with d_1 and d_2 taking values from $2, 5, 10, 20, \cdots, 100$. Then the dimensions of the concatenated feature representations are $4, 10, 20, 40, \cdots, 200$, respectively. For each value of d_1 and d_2, we use Rediscover for TFBS prediction for a given TF and assess the prediction performance using different evaluation metrics including AUPR and F_1 score. We then compare the prediction performance across the different values of d_1 and d_2 for each TF (with examples shown in Fig. S1), and also compare the distributions of performance (here we specifically use AUPR for comparison) across all the evaluated TFs between different choices of d_1 and d_2 (Fig. S2). We observed that for some TFs a small number of dimensions can have relatively high accuracy, while for other TFs the performance increases with the number of dimensions. The performance may remain relatively stable within a range of d_1 and d_2. The performance would decrease if a large number of dimensions are used, probably because of overfitting (Fig. S1). We found that 50 dimensions in each feature space (100 dimensions in total for the concatenated feature representation) can provide major gains in performance for many of the TFs. A large number of feature dimensions may lead to model overfitting. When applying Rediscover to new datasets, we may first compute the explained variance using specific numbers of components from the results of SVD, and choose d_1 and d_2 from the numbers that are associated with a relatively large explained variance and also not very high-dimensional in order to reduce model overfitting.

A.5 Application details of the compared methods

The *in silico* ChIP-seq library method [1] and our method used the curated CIS-BP motif collection [7, 8] and the motifmatchr package [9] for motif scanning, and share the same motif scanning results as input. We used p-value<5e-05 for motif identification, which is the threshold recommended by chromVAR [8]. We used the threshold 0.1 on the *in silico* TF binding score to select the peak-TF links. This threshold gives better F_1 score than the higher thresholds on the PBMC data. The authors recommended a threshold between 0.1 and 0.3 depending on the data analyzed.

GRaNIE was applied following the manual at https://grp-zaugg.embl-community.io/GRaNIE. The raw counts of ATAC-seq and RNA-seq data for each metacell were provided to GRaNIE as the authors recommended using their normalization procedures. The default parameters were used for the data normalization, with "Deseq_sizeFactor2" for the ATAC-seq data and "lima_quantile" for the RNA-seq data, respectively. We used the default parameter promoterRange=250000 to infer peak-gene connections, which represents searching for regulatory peaks within +/-250Kb of gene TSS. The default FDR thresholds to filter TF-peak connections and peak-gene connections to reconstruct the GRN are both 0.2. The *in silico* TF binding sites identified with the motif collection from HOCOMOCO [10] using PWMScan [11] for human genome (retrieved from https://difftf.readthedocs.io/en/latest) [12] were used, which are the authors' preferences. We originally used the default threshold FDR<0.2 to select the peak-TF links but we found the TF binding predicion performance by F_1 score is very low mainly due to low recall. We therefore adjusted the threshold to be FDR<0.3.

Pando [3] was applied following the manual at https://quadbiolab.github.io/Pando. Based on the descriptions in the paper, we used the intersections between the ATAC-seq peak loci and the provided sequence element annotations, including the PhastCons conserved elements [13] and the human cCREs (candidate cis-Regulatory Elements) derived from database of the ENCODE project [14] (https://screen.encodeproject.org) as the candidate regulatory regions. The exonic regions were excluded using the default parameter exclued_exons=TRUE in the initiate_grn function. Motif scanning was performed by Pando using the motifmatchr package [8] with the motif collection curated by the authors, which is a combination of the motifs from the JASPAR [15] and CIS-BP [7] databases and the inferred motifs. We used the default parameters in the infer_grn function for the GRN inference. Specifically, candidate regulatory regions up to 100Kb upstream of gene TSS and within the gene body were included. The generalized linear models were used for gene expression prediction. The peak-TF pair constituting an interaction term that has a significant estimated coefficient in the gene expression model is selected to present a peak-TF link. The default threshold is FDR adjusted p-value<0.05. However, the recall is very low using the default threshold. We therefore adjusts the threshold to p-value<0.1.

TRIPOD [4] was applied following the manual at https://github.com/yuchaojiang/TRIPOD. The union of the peak-TF-gene associations identified from the four tests (level 1 and level 2 tests for matching by TF expression or matching by peak accessibility) were used for analysis. The default threshold upstream=100, downstream=100 was used to include candidate regulatory peaks within +/-100Kb of gene TSS. Motif scanning was performed within TRIPOD through the CreateMotifMatrix function in Signac [16] using the motifmatchr package [9] with the JASPAR motif database [15] as the default choice. The peak-TF associations are retrieved from the inferred peak-TF-gene links.

We applied SCENIC+ [17] to the PBMC data for TFBS prediction and GRN inference following the manual at https://scenicplus.readthedocs.io/en/latest/. There are two sets of predicted peak-TF associations retrievable from SCENIC+: (i) prediction by the pycistarget package; (ii) peak-TF associations derived from the learned eRegulons (an eRegulon involves the prediced target genes and

target regions of a TF). We note (ii) as the predictions by SCENIC+(eRegulon). We observed that TFBS predictions in (i) have higher F_1 score than the predictions in (ii), which is probably because SCENIC+ only selects significant peak-gene and gene-TF associations to build the eRegulons. Part of the peak-TF links predicted by the pycistarget package may not be retained if the peak was not included in the eRegulon or the eRegulon was not estimated for the corresponding TF. We used the predictions in (i) for the comparison between our method and SCENIC+ as shown in Figure 2d. In (i), SCENIC+ performs TFBS prediction using motif enrichment analysis. Specifically, a TF is first associated to a group of peaks based on significance of enrichment, and then peaks with the TF motifs and the CRM (cis-regulatory module) scores above a threshold are selected from the group as the putative target peaks of the given TF. We used both cistarget algorithm and the DEM algorithm for TFBS predictions. The output of SCENIC+ contains he predicted target regions of each TF, but there is not one score associated with a peak-TF pair in the output of SCENIC+. Therefore AUPR is not applicable to compute. We use F_1 score for performance evaluation for SCENIC+.

TOBIAS [18] was applied following the manual at https://github.com/loosolab/TOBIAS/wiki. TOBIAS was developed for the bulk ATAC-seq data, requiring relatively high library size fragment file to perform bias correction and the footprint analysis. TOBIAS is not directly applicable to the single cell ATAC-seq data, which are generally sparse and noisy. To use TOBIAS in our analysis, we provided cell type-based pseudo-bulk ATAC-seq data to TOBIAS by aggregating the read alignment files of the cells in each cell type.

We computed the F_1 score for each method by comparing the predicted peak-TF links with the TF binding events supported by the ChIP-seq data for each analyzed TF. ($F_1 = \frac{2 \cdot presion \cdot recall}{precison + recall}$). We compute AUPR for REUNION based on the TF binding probabilities predicted by Rediscover. For the other methods, AUPR was calculated based on the retrievable peak-TF association scores, which are specifically the TF binding score estimated by TOBIAS, the *in silico* TF binding score estimated by the *in silico* ChIP-seq method, the FDR of peak-TF links estimated by GRaNIE, the lowest FDR-adjusted p-value of the interaction term for a peak-TF pair using Pando, the smallest adjusted p-value of the peak-TF-links involving the given peak-TF pair using TRIPOD, and the reported motif scores by motif scanning using the CIS-BP motif collection, respectively. For motif scanning with the other motif collections, binary motif detection results were provided by the corresponding multiome-based method where the scanning was performed as an internal step or external predicted TFBS annotations were utilized.

A.6 Model variant of Rediscover

In TF binding prediction on the PBMC data, we compared the TFBS prediction performance between REUNION and a model variant in which we omitted the clustering-guided pseudo-labeled training sample selection steps in Rediscover, which is noted as base model. Specifically, we used all the peaks with predicted TF binding by Rediscover (or a compared method, for example, motif scanning-based TFBS prediction or the *in silico* ChIP-seq method) as the pseudo positive training samples. From the peaks without predicted TF binding by Rediscover, we randomly selected a specific number of peaks as the pseudo negative training samples. Suppose we select n_1 pseudo positive training samples. We select $n_2 = r_1 n_1$ pseudo negative training samples, with r_1 as an adjustable ratio. We used $r_1 = 1.5$.

A.7 TF binding prediction performance evalutaion using ChIP-seq data

We downloaded ChIP-seq data from public databases including ENCODE data portal [19] and Cistrome DB [20, 21] to evaluate the TFBS prediction performance by our methods, in comparison with other

representative existing methods. For experiments that have multiple replicates, the TF binding prediction performance by REUNION and also by the other methods is usually similar between the replicates. We chose the data of the replicate with relatively higher overlapping between the ChIP-seq peaks and the ATAC-seq peaks. We have 67 ChIP-seq datasets for unique TF and cell type combinations after selection, involving 59 TFs and four cell types (B cell, T cell, Monocyte, and Macrophage). The information is shown below.

Cell type	TF	Data source
B cell	ATF2, ATF3, ATF7, BACH1, BACH2, BATF, BATF3, BCL6, CTCF, CUX1, E2F4, EBF1, EGR1, ETV6, FOS, FOXM1, GABPA, IKZF1, IRF2, IRF4, IRF5, JUND, MAX, MEF2A, MEF2B, MEF2C, MYC, NFIC, NR2C1, PAX5, PKNOX1, POU2F2, RELA, REST, RUNX3, SPI1, SPIB, STAT5A, STAT5B, TBP, TBX21, TCF12, TCF7, USF2, YY1, ZBTB33, ZEB1	Encode data portal; Cistrome DB
T cell	CEBPA, CEBPB, CTCF, FOXP3, GATA3, MYB, REST, RUNX1, STAT5B, TBX21, YY1, ZNF143	Cistrome DB
Monocyte	CEBPA, CTCF, IRF1, IRF8, SPI1, STAT1, VDR	Cistrome DB
Macrophage	SPI1	Cistrome DB

Table S1: The information of the downloaded ChIP-seq datasets.

A.8 Motif enrichment analysis in the peaks with or without the TF motif detected

Using Rediscover we perform TF binding prediction in the genome-wide peaks with or without the motifs of a given TF detected. In order to analyze the potential co-binding patterns of TF partners in the peaks predicted with TF binding, we performed motif enrichment analysis in two group of peaks that overlap with the TF ChIP-seq signals, which are: (1) the peaks with ChIP-seq signals and with TF binding motifs detected, and (2) the peaks with ChIP-seq signals but without TF motifs detected, and with TFBSs predicted by REUNION. The second group of peaks were hidden from identifying the peak-TF links for the given TF if we only use motif scanning results.

Suppose the given TF for which we predicted TF binding using REUNION is TF A. For each group of peaks as described above (noted as the target group), we merge all the other peaks as the alternative group. We counted the motif presence frequency of each TF in a peak in a group based on the motif scanning results, and performed Fisher's exact test between the target group and the alternative group to assess the statistical significance that the motif presence of a specific TF is enriched in the target group. The TF with significant enrichment of motif presence in the target group may have co-binding behaviors with the given TF A in the corresponding group of peaks.

A.9 Identifying potential new gene-TF associations based on TF binding predicted in peaks without the TF motif detected

By predicting TF binding in peaks without the motifs detected for the specific TF, we can establish new peak-TF associations and furthermore identify potential new gene-TF associations by linking the TF to the potential target gene through the peak-gene associations. There are two scenarios:

(1) The TF is already associated with the target gene through existing estimated peak-TF-gene links. Specifically, the TF has predicted binding sites in the peaks which contain the TF motif and are associated with the target gene by Unify. Predicted TF binding in the peaks without the TF motif detected adds new peak-TF-gene links, while the gene-TF links are the same.

(2) The TF is not associated with the target gene based on the estimation from Unify. If TF binding is predicted in a peak without the TF motif detected and the peak is possibly associated with

the target gene by distance proximity or the estimations from Unify, a new peak-TF-gene link can be established which connects the TF to the potential new target gene.

We focus on (2) in our analysis. For each TF with predicted TF binding in the peak loci without the corresponding TF motifs detected, we link the TF to the potential target genes through the possible candidate peak-gene links that were previously estimated by Unify before the step of utilizing the two designed score functions. For TF j, let S_j denote the set of new identified potential target genes of TF j, which are the genes not included in the peak-TF-gene associations estimated by Unify that involve the TF. Let $n_j = |S_j|$ denote the number of genes in S_j. For gene $g_k \in S_j$ ($k = 1, \cdots, n_j$), let V_k denote the set of candidate regulatory TFs of g_k which are estimated by Unify. After we apply Rediscover for TF binding prediction for TF j, the new candidate regulatory TFs of gene g_k is $\hat{V}_k = V_k \cup \{TFj\}$. We then estimate the potential regulatory associations between TF j and gene g_k by performing gene expression prediction using the TF expressions and comparing the prediction performance from using two sets of candidate regulatory TFs as the predictor variables, which are V_k and $\hat{V}_k$, respectively.

Specifically, we use the XGBoost regression model to predict expression of g_k using the expressions of TFs in V_k or $\hat{V}$, respectively, noted as prediction 1 and prediction 2. Since there is only one TF changed in the predictor variables between the two predictions, the gene expression prediction performance is expected to be without large change unless the new candidate TF j has strong effects on the gene expression prediction. If prediction 2 outperforms prediction 1 significantly, it provides evidence that there may exist regulatory associations between TF j and gene g_k. For each TF, we look for the potential new target genes for which the gene expression prediction accuracy measured by the Pearson correlation between the predicted and real gene expression is increased by at least 0.05 after adding the new TF as a predictor.

A.10 Identifying potential new TF interaction effects on chromatin accessibility

Predicting TF binding in the peaks without the TF motif detected may reveal potential interaction effects of TF partners on the chromatin accessibility. For peak i, let T_i and $T_i = T_i \cup \{\text{TF } j\}$ represent the estimated binding TFs before and after applying Rediscover to predict the binding sites of TF j, respectively. We compute two types of correlations using the TF set $\hat{T}_i$:

(1) The correlation between the peak accessibility and the expression of each single TF linked to the peak. Let ρ_{il} denote the correlation between peak i and TF l. We have $\{\rho_{il}|\text{TF } l \in \hat{T}_i\} = \{\rho_{il}|\text{TF } l \in T_i\} \cup \{\rho_{il}\}$.

(2) The correlation between the peak accessibility and the product of expressions of each pair of TFs linked to the peak. Let $\rho_{i,(l,l')}$ denote the correlation between peak i and the pair of TF l and TF l'. We have $\{\rho_{i,(l,l')}|\text{TF } l, l' \in T_i\} = \{\rho_{i,(l,l')}|\text{TF } l, l' \in T_i\}\{\rho_{i,(j,l')}|\text{TF } l' \in T_i\}$. The predicted new peak-TF links add $n_i = |T_i|$ new interactions between the given TF j and the binding TFs of peak i previously predicted by Unify.

Comparing between (1) and (2), for TF j and the new putative target peak i, we look for the possibilities that

$$\max_{l,l' \in T_i}\{\rho_{i,(l,l')})\} > \max_{l \in T_i}\{\rho_{il}\},$$
$$\max_{l,l' \in T_i}\{\rho_{i,(l,l')})\} = \max_{l' \in T_i}\{\rho_{i,(j,l')}\},$$

which presents: (i) there exist TF pairs of which the TF expression product has higher correlation with peak accessibility than single TFs; (ii) the TF pair which reaches the highest correlation in (1)

includes the new predicted binding TF j and a previously predicted binding TF l' for peak i.

Specifically, the existence of TF interaction effects on peak accessibility does not necessarily require that the peak-TF pair correlation for a given TF pair is larger than any peak-TF correlations for the individual TFs linked to the peak. Here we use the criteria as described above in order to identify relatively strong interaction effects to prioritize highly likely estimated new peak-TF associations. More TF pairs may be identified with potential interaction effects if we only require $\rho_{i,(l,l')} > \max\{\rho_{il}, \rho_{il'}\}$.

B Supplementary Figures

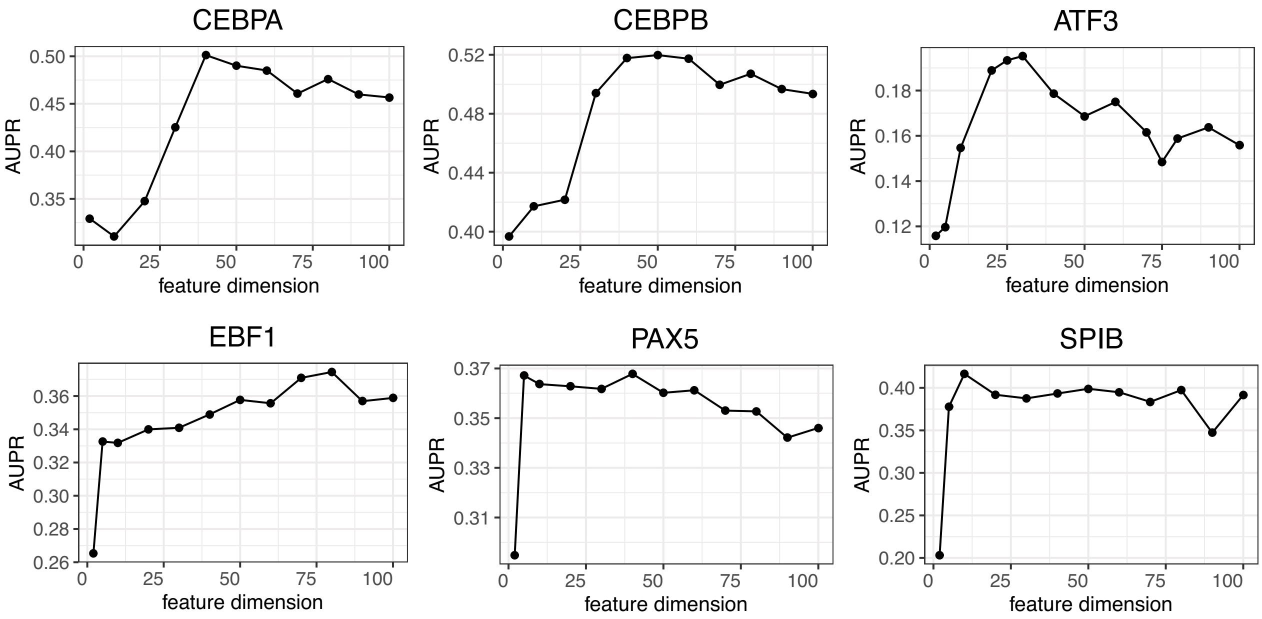

Figure S1: The change of TF binding prediction performance in genome-wide peaks evaluated by AUPR along with the different choices of the number of feature dimensions utilizing SVD in each feature space (the chromatin accessibility feature space and the sequence feature space, respectively) for the example TFs. The number of latent accessibility feature dimension d_1 and the number of latent sequence feature dimension d_2 take values from $2, 5, 10, 20, \cdots, 100$ synchronously, with the dimensions of the concatenated feature presentations being $4, 10, 20, 40, \cdots, 200$, respectively.

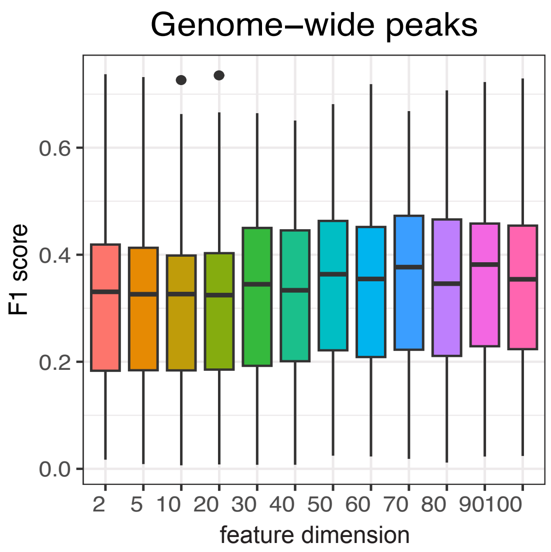

Figure S2: The boxplots of TF binding prediction performance in genome-wide peaks evaluated by AUPR across all the evaluated TFs at each choice of the number of feature dimensions utilizing SVD in each feature space (the chromatin accessibility feature space and the sequence feature space, respectively). The number of latent accessibility feature dimension d_1 and the number of latent sequence feature dimension d_2 take values from $2, 5, 10, 20, \cdots, 100$ synchronously, with the dimensions of the concatenated feature presentations being $4, 10, 20, 40, \cdots, 200$, respectively.

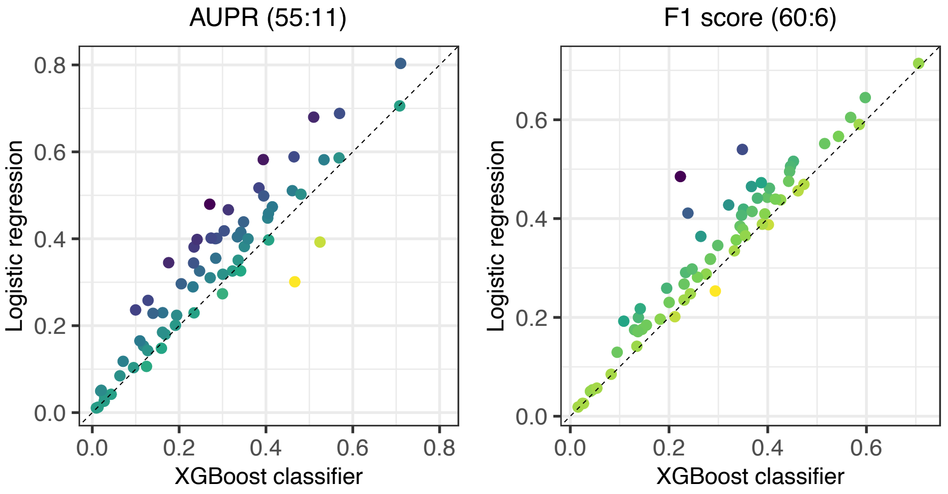

Figure S3: TF binding prediction performance comparison between using the logistic regression model and the XGBoost classifier as the prediction model in Rediscover for the evaluated TFs in the genome-wide peaks using AUPR (left) and F_1 score (right). In each subplot, the color of each dot is scaled according to the performance difference between using the logistic regression model and using the XGBoost classifier. The number of TF ChIP-seq datasets for which using the logistic regression model has higher or lower performance than using the XGBoost classifier by at least 0.001 appears above each subplot as $(n_1 : n_2)$.

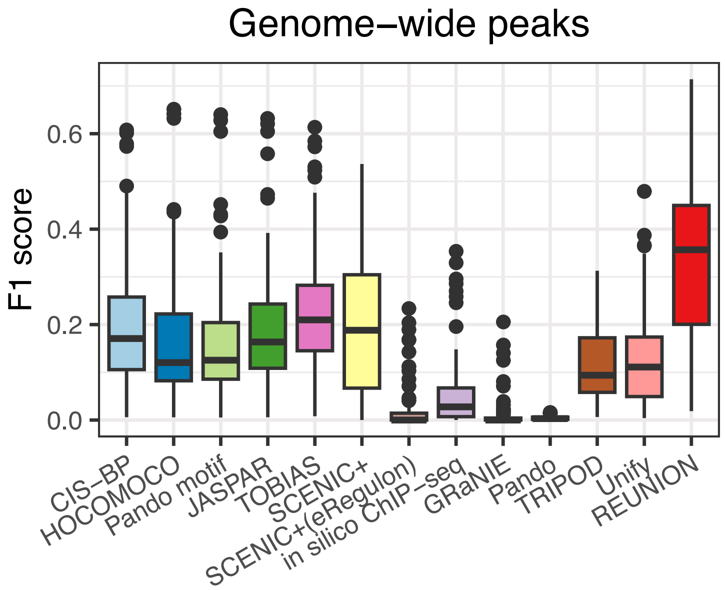

Figure S4: F_1 score distributions of TFBS predictions in genome-wide peaks (with or without the motif of a given TF detected) by the different methods for the evaluated TF ChIP-seq datasets.

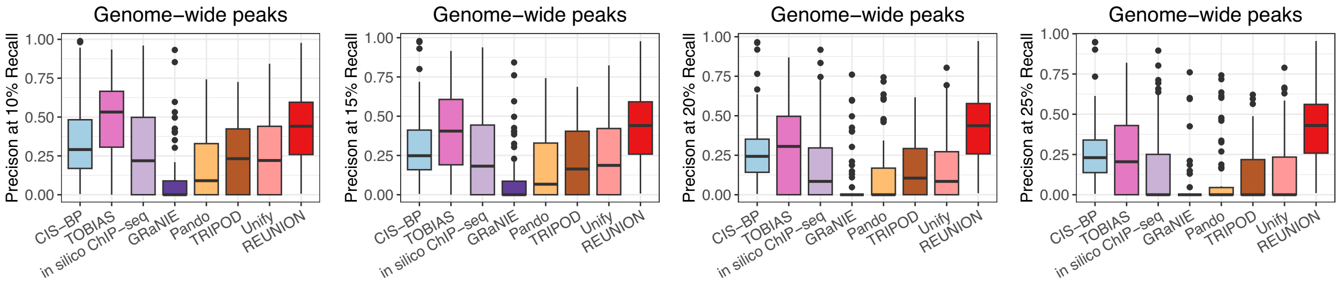

Figure S5: Performance comparison of TFBS prediction in genome-wide peaks between different methods evaluated by Precision at 10%, 15%, 20%, 25% Recall, respectively (from left to right).

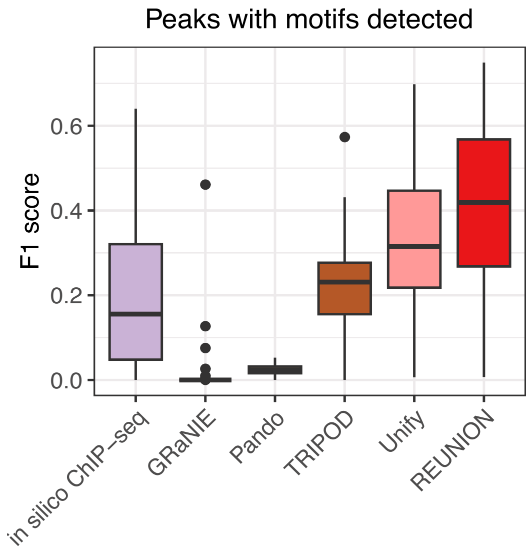

Figure S6: TF binding prediction performance comparison in peaks with the motifs of a given TF detected between the *in silico* ChIP-seq, GRaNIE, Pando, TRIPOD, Unify, and REUNION.

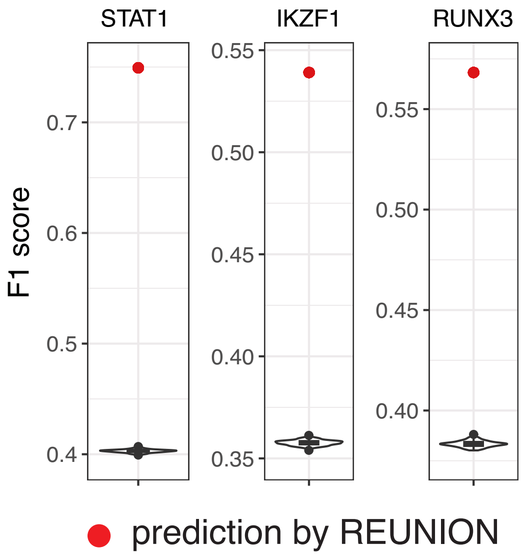

Figure S7: Examples showing the F_1 score of the TF binding prediction by REUNION in comparison with the distribution of F_1 scores (the boxplot) by the random sampling approach for TF binding prediction in the peak loci without the motif detected (Fig. 3h) for a give TF. The TF name is shown in the title of each subplot.

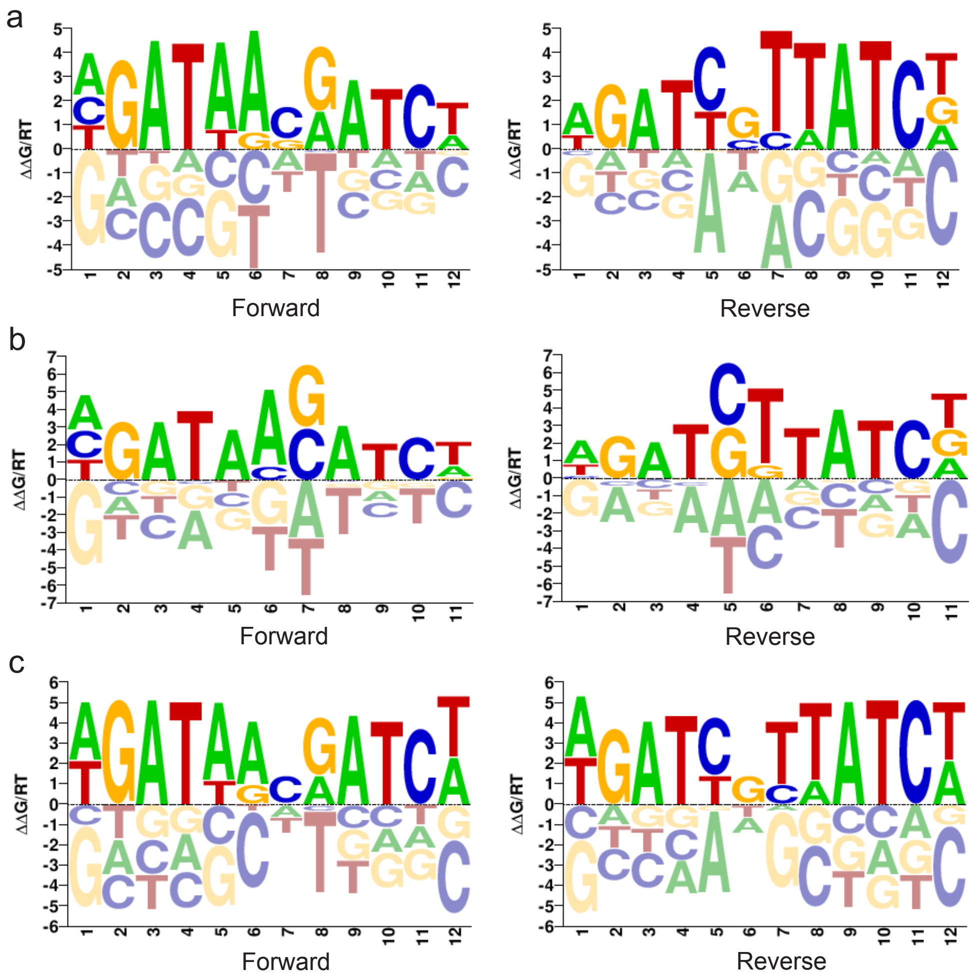

Figure S8: Examples of dimer motifs of Gata3 in the existing motif databases.

References

[1] Ricard Argelaguet, Tim Lohoff, Jingyu Gavin Li, Asif Nakhuda, Deborah Drage, Felix Krueger, Lars Velten, Stephen J Clark, and Wolf Reik. Decoding gene regulation in the mouse embryo using single-cell multi-omics. *bioRxiv*, pages 2022–06, 2022.

[2] Aryan Kamal, Christian Arnold, Annique Claringbould, Rim Moussa, Nila H Servaas, Maksim Kholmatov, Neha Daga, Daria Nogina, Sophia Mueller-Dott, Armando Reyes-Palomares, et al. Granie and granpa: inference and evaluation of enhancer-mediated gene regulatory networks. *Molecular Systems Biology*, page e11627, 2023.

[3] Jonas Simon Fleck, Sophie Martina Johanna Jansen, Damian Wollny, Fides Zenk, Makiko Seimiya, Akanksha Jain, Ryoko Okamoto, Malgorzata Santel, Zhisong He, J Gray Camp, et al. Inferring and perturbing cell fate regulomes in human brain organoids. *Nature*, 621(7978):365–372, 2023.

[4] Yuchao Jiang, Yuriko Harigaya, Zhaojun Zhang, Hongpan Zhang, Chongzhi Zang, and Nancy R Zhang. Nonparametric single-cell multiomic characterization of trio relationships between transcription factors, target genes, and cis-regulatory regions. *Cell Systems*, 13(9):737–751, 2022.

[5] Jack Edmonds. Paths, trees, and flowers. *Canadian Journal of mathematics*, 17:449–467, 1965.

[6] Silvio Micali and Vijay V Vazirani. An o (v— v— c— e—) algoithm for finding maximum matching in general graphs. In *21st Annual Symposium on Foundations of Computer Science (sfcs 1980)*, pages 17–27. IEEE, 1980.

[7] Matthew T Weirauch, Ally Yang, Mihai Albu, Atina G Cote, Alejandro Montenegro-Montero, Philipp Drewe, Hamed S Najafabadi, Samuel A Lambert, Ishminder Mann, Kate Cook, et al. Determination and inference of eukaryotic transcription factor sequence specificity. *Cell*, 158 (6):1431–1443, 2014.

[8] Alicia N Schep, Beijing Wu, Jason D Buenrostro, and William J Greenleaf. chromvar: inferring transcription-factor-associated accessibility from single-cell epigenomic data. *Nature methods*, 14(10):975–978, 2017.

[9] Alicia N Schep. motifmatchr: Fast motif matching in R. *doi:10.18129/B9.bioc.motifmatchr*, 2023.

[10] Ivan V Kulakovskiy, Ilya E Vorontsov, Ivan S Yevshin, Ruslan N Sharipov, Alla D Fedorova, Eugene I Rumynskiy, Yulia A Medvedeva, Arturo Magana-Mora, Vladimir B Bajic, Dmitry A Papatsenko, et al. Hocomoco: towards a complete collection of transcription factor binding models for human and mouse via large-scale chip-seq analysis. *Nucleic acids research*, 46(D1): D252–D259, 2018.

[11] Giovanna Ambrosini, Romain Groux, and Philipp Bucher. Pwmscan: a fast tool for scanning entire genomes with a position-specific weight matrix. *Bioinformatics*, 34(14):2483–2484, 2018.

[12] Ivan Berest, Christian Arnold, Armando Reyes-Palomares, Giovanni Palla, Kasper Dindler Rasmussen, Holly Giles, Peter-Martin Bruch, Wolfgang Huber, Sascha Dietrich, Kristian Helin, et al. Quantification of differential transcription factor activity and multiomics-based classification into activators and repressors: diffTF. *Cell reports*, 29(10):3147–3159, 2019.

[13] Adam Siepel, Gill Bejerano, Jakob S Pedersen, Angie S Hinrichs, Minmei Hou, Kate Rosenbloom, Hiram Clawson, John Spieth, LaDeana W Hillier, Stephen Richards, et al. Evolutionarily conserved elements in vertebrate, insect, worm, and yeast genomes. *Genome research*, 15(8): 1034–1050, 2005.

[14] Jill E Moore, Michael J Purcaro, Henry E Pratt, Charles B Epstein, Noam Shoresh, Jessika Adrian, Trupti Kawli, Carrie A Davis, Alexander Dobin, et al. Expanded encyclopaedias of dna

elements in the human and mouse genomes. *Nature*, 583(7818):699–710, 2020.

[15] Oriol Fornes, Jaime A Castro-Mondragon, Aziz Khan, Robin Van der Lee, Xi Zhang, Phillip A Richmond, Bhavi P Modi, Solenne Correard, Marius Gheorghe, Damir Baranašić, et al. Jaspar 2020: update of the open-access database of transcription factor binding profiles. *Nucleic acids research*, 48(D1):D87–D92, 2020.

[16] Tim Stuart, Avi Srivastava, Shaista Madad, Caleb A Lareau, and Rahul Satija. Single-cell chromatin state analysis with signac. *Nature methods*, 18(11):1333–1341, 2021.

[17] Carmen Bravo González-Blas, Seppe De Winter, Gert Hulselmans, Nikolai Hecker, Irina Matetovici, Valerie Christiaens, Suresh Poovathingal, Jasper Wouters, Sara Aibar, and Stein Aerts. Scenic+: single-cell multiomic inference of enhancers and gene regulatory networks. *Nature Methods*, 20(9):1355–1367, 2023.

[18] Mette Bentsen, Philipp Goymann, Hendrik Schultheis, Kathrin Klee, Anastasiia Petrova, René Wiegandt, Annika Fust, Jens Preussner, Carsten Kuenne, Thomas Braun, et al. Atac-seq footprinting unravels kinetics of transcription factor binding during zygotic genome activation. *Nature communications*, 11(1):4267, 2020.

[19] Yunhai Luo, Benjamin C Hitz, Idan Gabdank, Jason A Hilton, Meenakshi S Kagda, Bonita Lam, Zachary Myers, Paul Sud, Jennifer Jou, Khine Lin, et al. New developments on the encyclopedia of dna elements (encode) data portal. *Nucleic acids research*, 48(D1):D882–D889, 2020.

[20] Rongbin Zheng, Changxin Wan, Shenglin Mei, Qian Qin, Qiu Wu, Hanfei Sun, Chen-Hao Chen, Myles Brown, Xiaoyan Zhang, Clifford A Meyer, et al. Cistrome data browser: expanded datasets and new tools for gene regulatory analysis. *Nucleic acids research*, 47(D1):D729–D735, 2019.

[21] Shenglin Mei, Qian Qin, Qiu Wu, Hanfei Sun, Rongbin Zheng, Chongzhi Zang, Muyuan Zhu, Jiaxin Wu, Xiaohui Shi, Len Taing, et al. Cistrome data browser: a data portal for chip-seq and chromatin accessibility data in human and mouse. *Nucleic acids research*, page gkw983, 2016.
